# Mapping tropical forest aboveground biomass using airborne SAR tomography

**DOI:** 10.1038/s41598-023-33311-y

**Published:** 2023-04-17

**Authors:** Naveen Ramachandran, Sassan Saatchi, Stefano Tebaldini, Mauro Mariotti d’Alessandro, Onkar Dikshit

**Affiliations:** 1 Department of Civil Engineering, Indian Institute of Technology Kanpur, Kanpur, 208016 India; 2 Jet Propulsion Laboratory (JPL), California Institute of Technology, Pasadena, CA 91125 USA; 3 Dipartimento di Elettronica, Informazione e Bioingegneria, Politecnico di Milano, Milan, 20133 Italy

**Keywords:** Forestry, Forest ecology, Climate and Earth system modelling, Forestry

## Abstract

Mapping tropical forest aboveground biomass (AGB) is important for quantifying emissions from land use change and evaluating climate mitigation strategies but remains a challenging problem for remote sensing observations. Here, we evaluate the capability of mapping AGB across a dense tropical forest using tomographic Synthetic Aperture Radar (TomoSAR) measurements at P-band frequency that will be available from the European Space Agency’s BIOMASS mission in 2024. To retrieve AGB, we compare three different TomoSAR reconstruction algorithms, back-projection (BP), Capon, and MUltiple SIgnal Classification (MUSIC), and validate AGB estimation from models using TomoSAR variables: backscattered power at 30 m height, forest height (FH), backscatter power metric (Q), and their combination. TropiSAR airborne campaign data in French Guiana, inventory plots, and airborne LiDAR measurements are used as reference data to develop models and calculate the AGB estimation uncertainty. We used univariate and multivariate regression models to estimate AGB at 4-ha grid cells, the nominal resolution of the BIOMASS mission. Our results show that the BP-based variables produced better AGB estimates compared to their counterparts, suggesting a more straightforward TomoSAR processing for the mission. The tomographic FH and AGB estimation have an average relative uncertainty of less than 10% with negligible systematic error across the entire biomass range (~ 200–500 Mg ha^−1^). We show that the backscattered power at 30 m height at HV polarization is the best single measurement to estimate AGB with significantly better accuracy than the LiDAR height metrics, and combining it with FH improved the accuracy of AGB estimation to less than 7% of the mean. Our study implies that using multiple information from P-band TomoSAR data from the BIOMASS mission provides a new capability to map tropical forest biomass and its changes accurately.

## Introduction

Forest ecosystem structure and AGB play a significant role in the global carbon cycles^1–3^. Accurate quantification of forest three-dimensional (3D) structure, biomass, and its dynamics over a regional and global scale are essential to understanding anthropogenic carbon emission and its climate response^3,4^. However, the heterogeneity in forest structure results in the variation of biomass stocks across the landscape that may introduce significant uncertainty in calculating emissions and removals of carbon from disturbance and recovery of forests^5,6^. A variation of forest structure and AGB is also reflected in forest dynamics over time, suggesting additional difficulty in quantifying forest AGB over time. Measurements that can provide spatial variations of forest structure and AGB over time are considered critical for reducing the uncertainty of the terrestrial carbon cycle^3,7,8^. Mapping AGB and its changes are more challenging in humid tropical forests because of the diversity of tree species, the complexity of structure due to tree size and shape, and temporal dynamics due to natural and human-induced changes^9^. Furthermore, these ecosystems are a significant carbon pool in AGB and contribute more than two-thirds of global terrestrial fluxes^10,11^.

Satellite remote sensing has been utilized for monitoring and mapping forests, including AGB, for several years^12–15^. The ability of Synthetic Aperture Radar (SAR) sensors to penetrate through clouds and forests, along with its sensitivity to dielectric and geometric properties of the target^16,17^, provides unique information, making it a viable tool for forestry. The SAR signal’s sensitivity and level of saturation (decrease in sensitivity of SAR signals to AGB values beyond a particular AGB value) to AGB varies according to its wavelength^18–20^. The lower frequency (L- and P-band) SAR can penetrate deep into the forest, interacting with its components, such as branches, trunks, etc.^21,22^, and hence better correlated to AGB over dense tropical forests. In addition, its ability to provide polarimetric and interferometric data helps to characterize the forest 3D structure using PolInSAR or TomoSAR approaches^21,23–25^, improving AGB estimates. Using multi-baseline SAR interferometric measurements to develop TomoSAR observations of forest structure has been one of the most promising remote sensing techniques to map AGB and its changes through time^25–28^. TomoSAR measurement acquires data at different view angles by changing spatial baselines to form a data stack containing multiple SAR images of the same area. The data stack is then focused via digital signal processing to produce a collection of voxels representing the reflectivity in three dimensions, thus allowing direct imaging of the interior of the forest canopy^29^. Different approaches have been adopted to inverse the reflectivity profile from multi-baseline data, the simplest of which is via the back-projection (BP) approach^30–33^. However, this approach is constrained by the limited vertical resolution of the profile, which is related to the total TomoSAR aperture length (${L}_{\mathrm{tomo}}$). Several super-resolution spectral estimation approaches used for TomoSAR image processing in the literature^16,24,25^ resolve the vertical backscatter power with higher resolution but suffers from poor radiometric accuracy.

However, the P-band SAR data or TomoSAR measurements from the spaceborne sensor are still unavailable. The ESA’s Earth Explorer’s BIOMASS spaceborne sensor is expected to be launched in 2024 with the first P-band SAR sensor^34,35^. The sensor will provide the first 3D measurements of the global forest using TomoSAR observation for a period of fourteen months after the initial commissioning phase. Since the data is acquired at P-band, it could also assist first mapping of terrain topography, even in dense forests^36,37^. Apart from these observations, the sensor will also operate in PolInSAR mode, allowing us to measure FH^35,38^. The mission is expected to produce global AGB and FH maps at a resolution of 4-ha (200 m × 200 m). As part of the calibration and validation activities of the BIOMASS mission, numerous airborne campaigns have been carried out demonstrating the capability of PolInSAR and TomoSAR techniques in estimating FH and/or AGB in boreal and tropical forests^25,27,28,39–41^. The results of these measurements have demonstrated that the TomoSAR backscatter power at linear polarizations and at an average height of 30 m above ground is strongly correlated with the AGB without any indication of saturation across the entire biomass range (up to 500 Mg ha^−1^)^20,24^. The 30 m height corresponded to the total backscatter power within a layer between 20 to 40 m (the tomogram resolution was about 20 m for TropiSAR), which is expected to have about 35% to 40% of the total AGB of the forest as predicted with the TROLL ecological model^42^. A similar relationship was found using the vertical profile from airborne LiDAR scanning (ALS) data relating AGB to the total canopy area of larger trees at approximately 30 m (25–32 m) above ground^43^. However, the TomoSAR-biomass relationship’s robustness and the AGB estimation uncertainty depended on the ground contribution. At P-band, it was observed that the SAR signal reaches the ground, and a considerable backscattering from the ground was observed in all polarization channels (but at different levels)^27,44^. Depending on the terrain slope, the double-bounce (canopy-ground or trunk-ground interaction) scattering is dominated if the terrain is flat, whereas,  in contrast, over sloped terrain, single-bounce scattering is visible^44^. One approach was to use a precise Digital Terrain Model (DTM) to minimize the terrain’s contribution and fine-tune the backscatter AGB model^28,39^. However, the lack of available precise and high-resolution DTM globally made this approach less effective over regions with high slopes. A recent approach estimated DTM directly from TomoSAR measurements comparable to LiDAR DTM in terms of precision^36,37^ and can be adopted for the same. Another solution is to decompose the TomoSAR backscatter contribution into ground and volume components^45^ and then correlate the volume contribution to AGB. Further, recent studies based on removing ground-level contribution from interferometric pairs^46–49^ have been shown to improve the results but fail to achieve the same level of accuracy as the 30 m TomoSAR backscatter intensity.

Although the airborne datasets have demonstrated the excellent performance of TomoSAR data for AGB estimation, retrieving AGB from spaceborne data over various regimes still poses challenges. (a) the spaceborne sensor will acquire data at a coarser resolution with a large coverage resulting in increased variation of forest and environmental characteristics within the resolution cell. For example, the variation of moisture and/or forest species and/or terrain condition may vary due to the increased size of the resolution cell. (b) Further, the viewing geometry of spaceborne sensors will have a narrow incidence angle, causing deeper penetration, resulting in more ground contribution effect than in airborne sensors. (c) although the backscattered power at the 30 m layer has shown the highest correlation with AGB over the tropical forest dataset^28^, the 30 m height doesn’t geometrically and physically justify the canopy height of global forest biomes and the related biomass content. For example, regions with smaller tree heights (say 15 m) or very high tree heights (say 50 m) will not necessarily have a good correlation for AGB with backscatter power at a 30 m layer. Further, the limitation of ground AGB data to find the fixed height, for a region, with the best correlation to AGB is constrained. Hence, addressing and validating backscattered power quantities other than a fixed height layer is essential. Recently, a new study integrated intensity along vertical profiles between 10 and 30 m heights as the volume contribution for estimating AGB, pointing to the role of vertical resolution in reducing the bias during AGB retrieval^40,41^. However, the effectiveness of this fixed height approach may constrain its practicability across regimes. Hence, a quantity that uses an adaptive approach depending upon the forest heights of the regimes has to be adopted. (e) The development of AGB estimate from the TomoSAR study has been widely dependent on the ground and LiDAR AGB and FH products. The lack of distribution of ground data around the globe constrains this approach. The availability of GEDI data allows us to overcome this limitation. However, recent studies using GEDI data have also shown a higher level of uncertainty in AGB estimates over multi-layered forests^50^. Hence, the validity of using LiDAR AGB products has to be assessed.

In view of these, our study attempted to predict AGB over the dense tropical forest by addressing the following questions. (a) can multi-polarization information enhance the accuracy of AGB estimates? (b) can the combination of backscattered power at 30 m height and tomographic height improve the AGB estimates counteracting the drawbacks of each other? (c) can an adaptive backscattered power quantity be derived to account for variation in FH over different regimes and with minimal dependency on reference AGB data? (d) does LiDAR AGB provide better accuracy compared to TomoSAR AGB estimates? and (e) Can the LiDAR AGB data be used to calibrate/validate TomoSAR AGB models? The experimental data for this study is from airborne P-band observations and in situ measurements acquired over the Paracou test site in French Guiana during ESA’s TropiSAR 2009 campaign^22^.

## Materials and methods

### Study area

**Figure 1:**
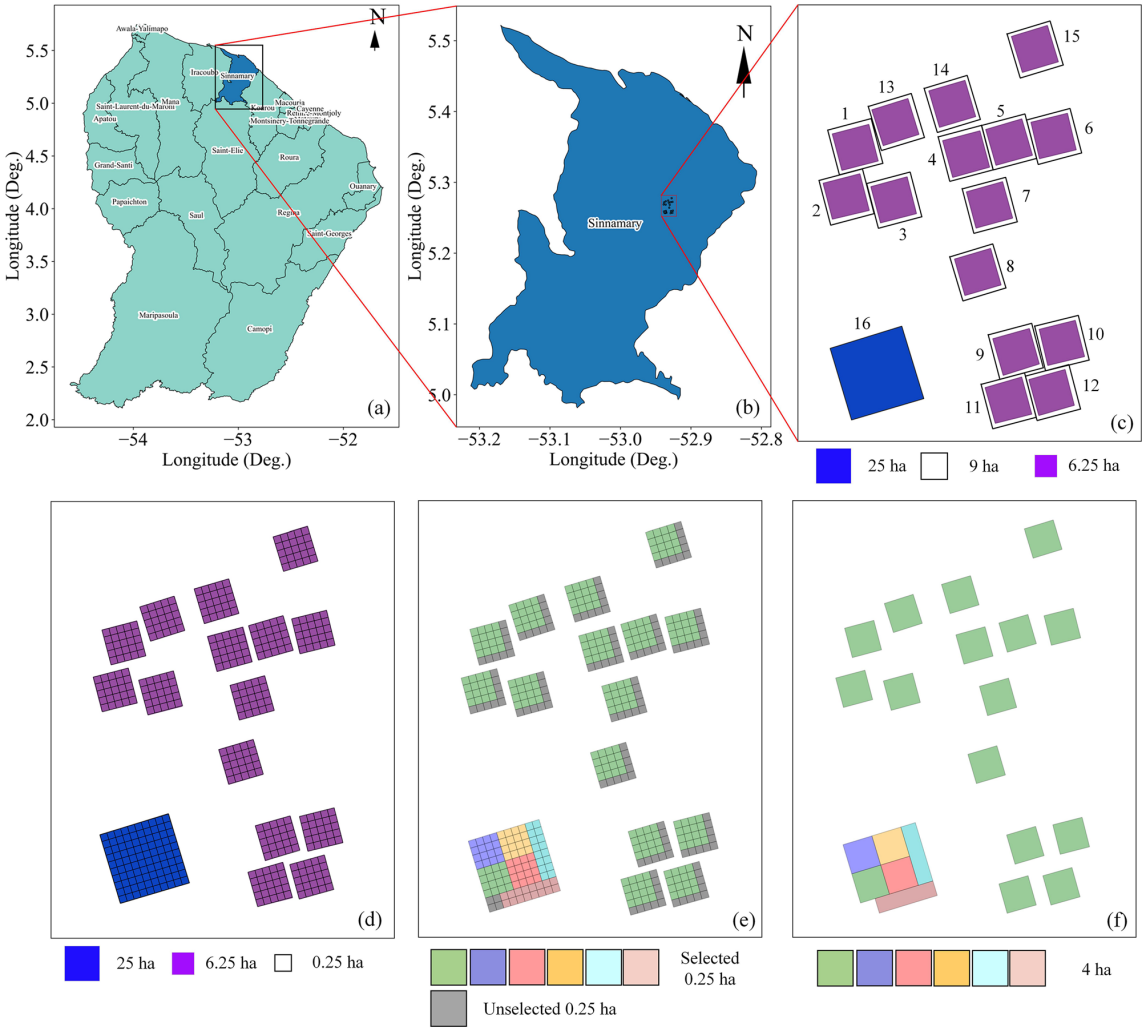
The locations of the Paracou test sites, French Guiana. (**a**,**b**) are based on the shapefiles from OCHA Field Information Services Section (FISS)^53^, and (**c**,**d**) are generated from the shapefiles discussed in Labriere et al.^54^ and the dataset can be obtained from^55^ (**e**) 0.25-ha plots selected for 4-ha plot generation (**f**) generated 4-ha plots. This Figure is generated using python 3.7.4.^56^.

The experimental site (5°18′ N,52° 55′ W) is located in the moist tropical forests of Paracou, 40 km west of Kourou and 12 km east of Sinnamary village, in French Guiana (Fig. 1a,b). The study area is hilly, with terrain heights varying from 5 to 50 m above mean sea level, with average annual precipitation of 3041 mm. The flora in the site is primarily dominated by tree families of Caesalpiniaceae, Lecythidaceae, Chrysobalanaceae, and Sapotaceae^51^. In 1984, twelve 9-ha (300 m × 300 m) were established, which were divided into three homogenous blocks^52^. Between 1986 and 1988, they underwent three types of silvicultural treatments (applied to one plot within each block and the last one as a control plot) with different intensities based on the randomized block design. For the details of the silvicultural treatments, one can refer to^52^. Later, three more 9-ha (300 m × 300 m) and 25-ha (500 m × 500 m) plots were established in 1990 and 1991/1992, respectively. Since 1986, these sixteen plots (12 + 3 + 1) have been regularly surveyed, and trees above 10 cm in diameter, along with species information, were mapped (Fig. 1c). However, over the fifteen 9-ha plots, the tree characteristic was monitored only in a core area covering 6.25-ha (250 m × 250 m), as shown in Fig. 1c. This periodic monitoring of the site forms a solid forest research database.

### Inventory data

As a part of the preliminary activities supporting the BIOMASS mission, the ESA conducted TropiSAR 2009 campaign and collected ground, airborne SAR, and LiDAR data over two sites, Nouragues and Paracou, in French Guiana^22^. The Office National d'Etudes et de Recherches Aérospatiales (ONERA) acquired SAR data, while The Evolution and Diversité Biologique laboratory (EDB) and Centre de coopération internationale en recherche agronomique pour le développement (CIRAD) where responsible for measuring reference ground and LiDAR data. ESA provides the standardized ground and LiDAR datasets at 0.25-ha resolution (50 m × 50 m). At Paracou, the 6.25-ha plots were divided into twenty-five 50 m × 50 m, and 25-ha plots were divided into hundred 50 m × 50 m plots (Fig. 1d). Labriere et al.^54^ estimated ground AGB values, which use height derived from the local H:D relationship, over the 0.25-ha were used as a reference AGB. For this work, analogous to BIOMASS products (AGB and tree height) resolution, the 4-ha plots (200 m × 200 m) were generated by aggregating the corresponding values from 0.25-ha plots. The 0.25-ha plots used to generate 4-ha plots are shown in Fig. 1e. The aggregated ground and LiDAR AGB and tree height values estimated at the 4-ha resolution (Fig. 1f) were used in this study. The average tree height was 27 m, with a mean biomass value of 340 Mg ha^−1^ over both 4-ha and selected 0.25-ha plots. However, the standard deviation was much higher (71.08 Mg ha^−1^ and 3.22 m) for 0.25-ha plots compared to the 4-ha plots (44.64 Mg ha^−1^ and 2.57 m) (Supplementary Table S1). The distribution of 0.25-ha and 4-ha AGB and tree heights are shown in Supplementary Fig. S1.

### Airborne SAR data

The TomoSAR image stack collected over the study site consists of six fully polarimetric single-look complex (SLC) data acquired at P-band during TropiSAR 2009 campaign. The sensor operates at a central frequency of 397.5 MHz with 125 MHz bandwidth. Flying at an altitude of 4014 m, the SAR sensor covers a swath width of 5 km with incidence angles between 25° and 60°. The TomoSAR stack is acquired by shifting the trajectory vertically by ~ 15 m below the reference track and a pixel spacing of 1 m and 1.245 m in slant range and azimuth direction, respectively. Further information about the campaign and datasets can be found in^22^. The configuration of P-band ONERA used during the campaign is shown in Table 1.

**Table 1:** Description of the TomoSAR Dataset acquired using P-band ONERA SETHI sensor.

Geometry	
Flight ground altitude (m)	4014
Aircraft speed (m/s)	120
Waveform	
Band/ Mode	P/Full-polar
Incidence angle range (deg.)	24–62
Frequency range (MHz)	260–460
PRF (KHz)	2.5
Wavelength (cm)	0.75
SLC image	
Pixel size (rg/az) (m)	1.0/1.245
Resolution (rg/az) (m)	1.2/ 1.5
No. of passes	6
Baseline range (m)	76.2

### Airborne LiDAR data

The ALS data from the study site was acquired under the project GUYAFOR by CIRAD. The laser operated at a wavelength of 0.9 μm with a pulse density of approximately 4 m^-2^ and a footprint size of approximately 45 cm at ground level. The raw data were processed, and the final canopy height model (CHM) and digital elevation model (DEM) were sampled at 1 m × 1 m pixels in size^57^. However, the LiDAR data is not available for the entire extent of the SAR image. The LiDAR-derived height matrices over these plots were used to estimate the LiDAR AGB product^54^.

### Methods

**Figure 2:**
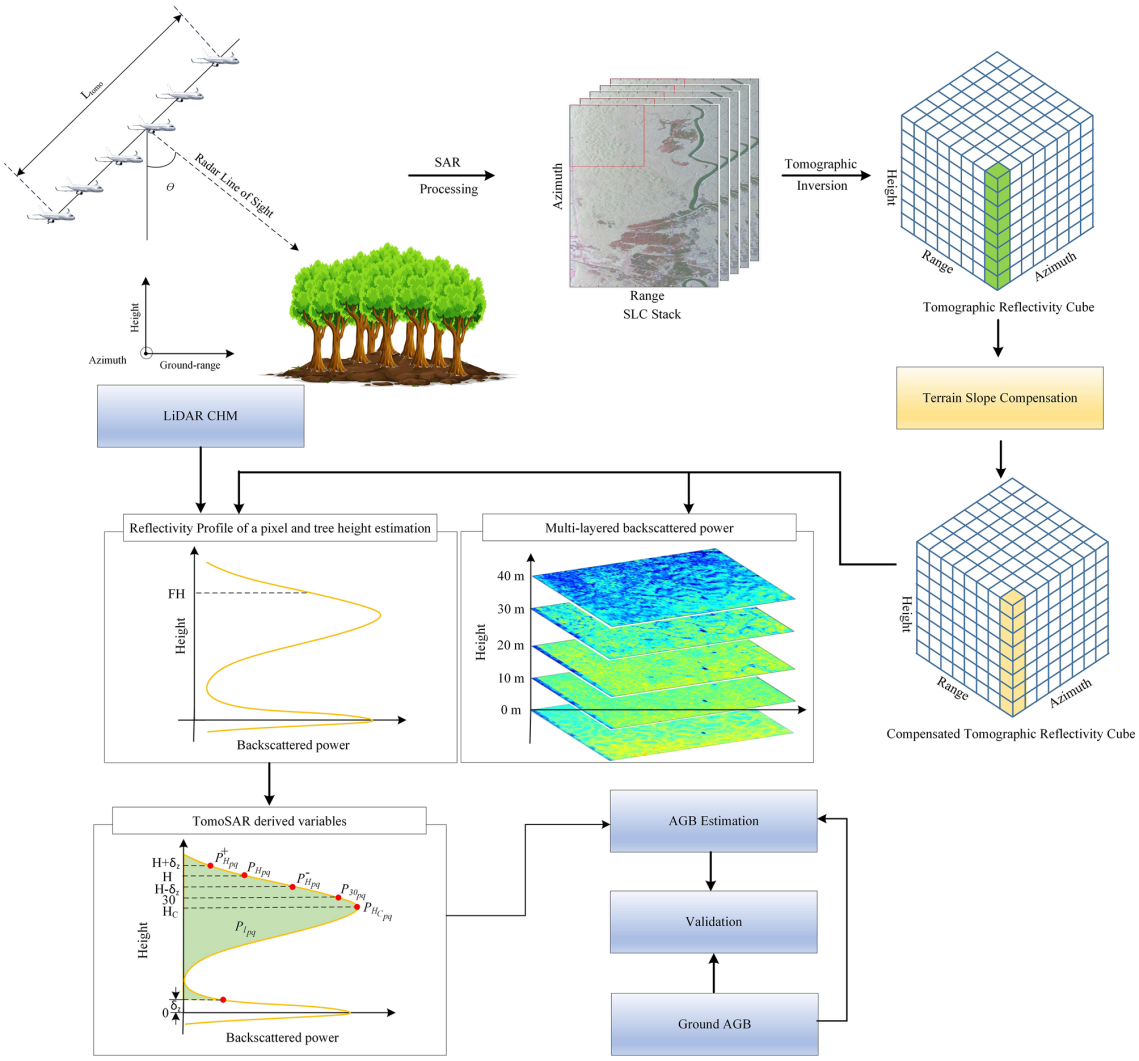
Overview of the methodology followed in this research. This Figure is generated using Inkscape 1.2.2^58^. The images of tree, ground and airplane in figure are used from^59–61^, respectively.

The details on the TomoSAR processing, generation of TomoSAR cube, forest profiles, and tree height estimation over the same dataset have been discussed in^47^, to which readers can refer a detailed understanding of TomoSAR processing. Here, we only summarize these steps, as the main focus of this study is to estimate AGB from the TomoSAR cube reconstructed and its derived variables. The overall methodology is shown in Fig. 2.

The focused and co-registered multi-baseline SAR data stack was phase-calibrated using the Phase Centre Double Localization approach^21^. Later, the interferometric coherence was calculated, and the TomoSAR reflectivity cube was reconstructed using BP^25^, Capon^62^, and MUSIC^62^ estimators (summarized in Supplementary Method S1). The reconstructed TomoSAR reflectivity cube was converted from radar geometry to ground geometry by interpolating the backscattered power from slant range-cross range geometry to the ground-elevation geometry with the knowledge of platform positions and terrain topography. Finally, the TomoSAR reflectivity cube was corrected for the influence of terrain relief^25,28,39^ and is given by

1 $${P}_{m}^{s}\left(x,g, z\right)={P}_{m}\left(x,g, z\right).\mathit{sin}\left(\theta -\alpha \right)$$

Here, ${P}_{\mathrm{m}}^{s}\left(x,g, z\right)$ is the slope compensated TomoSAR reflectivity within the azimuth-ground range resolution cell ($x,g$) at height layer $z$ from the ground level using estimator $m$, $\theta$ is the radar incidence angle $\alpha$ denotes the local slope in ground range geometry, and $m$ represents the reconstruction algorithm ($m$ can be BP or Capon or MUSIC). The FH was estimated from the TomoSAR using the approach adopted by^27^ (summarized in Supplementary Method S2).

Further, in this study, we derive the TomoSAR power metric (Q) based on vertical resolution, retrieved FH, and phase center, as defined below in Eq. (2), to estimate and validate a variable that can substitute for TomoSAR backscatter power at 30 m. To provide simplified notation, ${P}_{m}^{s}$ from Eq. (1) is written as ${P}_{pq}$ in Eq. (2), where $P={P}_{m}^{s}$ and $p,q$ represents transmitted and received polarization.

2 $$\left.\begin{array}{l}{P}_{{H}_{pq}}= {P}_{pq}\left(H,x,g\right)\quad (Q1)\\ {{P}_{{H}_{pq}^{-}}=P}_{pq}\left(H-0.5{*\delta }_{z}, x,g\right) \quad (Q2)\\ \begin{array}{c}{{P}_{{H}_{pq}^{+}}=P}_{pq}\left(H+0.5{*\delta }_{z}, x,g\right)\quad (Q3)\\ {P}_{{I}_{pq}}=\underset{0.5{*\delta }_{z}}{\overset{H}{\int }}{P}_{pq}\left(x,g\right)dz \quad (Q4)\\ {P}_{{{H}_{c}}_{pq}}={P}_{pq}\left({H}_{c},x,g\right)\quad (Q5)\end{array}\end{array}\right\}$$

Here, ${P}_{Hpq}, {P}_{{H}_{pq}^{-}, }{ , {P}_{{H}_{pq}^{+}}, P}_{{I}_{pq}} \mathrm{and }{P}_{{{H}_{c}}_{pq}}$ are the reflectivity of the TomoSAR cube at tree height (Q1), tree height minus half vertical resolution (Q2), tree height plus half vertical resolution (Q3), integrated reflectivity from half vertical resolution from ground to tree height (Q4) and phase center (Q5)*.* It is to be noted that the metric Q4, the lower cut-off of half of the vertical resolution from the ground, is selected to minimize the limitation due to the ground and double bounce scattering that degrades the correlation between the integrated power and AGB and is integrated up to estimated tree height values to adopt for representative heights rather than just the 30 m layer. Thus, this metric provides a much better representation of the physical structure than a single-layer metric.

### AGB model parameterization and validation

Previous studies^25,28,40,41,46^ have validated the potential of the backscattered power at the 30 m layer to estimate AGB using the BP estimator. However, this paper focuses on the AGB estimation from three variables: backscattered power at the 30 m layer, FH and Q (shown in Fig. 2.), and their combination, estimated from BP, Capon, and MUSIC. The model notations and quantity used are summarised in Table 2, and the corresponding AGB models are detailed in Supplementary Tables S4–S10. The backscattered power at the 30 m layer-based AGB models is classified into (a) single polarization AGB models (*P1-P15*) and (b) multi-polarimetric AGB models (*P16-P34*). Past studies have used different assumptions to explore the relationship between AGB and conventional SAR backscatter data^18,19,63–65^. Further, it was observed that combining information at different polarization^5,65–67^ and also with other parameters^5,68,69^, such as tree height, improved the AGB estimates in the case of conventional SAR backscatter data. Previous TomoSAR studies used a linear relationship assumption between AGB and backscattered power at 30 m of TomoSAR cube^25,28^. Hence, to explore further possibilities, we test the relationship between the backscattered power at the 30 m layer of the TomoSAR cube with the field AGB using linear, pure quadratic, exponential, power, and sigmoid forms. The power and sigmoid forms utilized linear values of backscattered power at the 30 m layer, whereas the other forms used log values. Similarly, we tried to establish a relationship between tomographic FH and the field AGB using the same assumptions (*H1–H15*). However, all the forms utilized linear values of FH. Further, the tomographic FH is combined with the single (*C1–C9*) and multi-polarization (*C10-C18*) backscattered power at the 30 m layer to calibrate and validate the AGB model. TomoSAR power metric-based (*QP1–QP24*) and the integrated TomoSAR power metric and tomographic FH (*QC1–QC18*) models are also evaluated. Once the average value of TomoSAR variables is extracted over the 4-ha plots, the AGB models are calibrated/validated using the hold-out validation approach by randomly selecting 75 percent as training and 25 percent as testing data iteratively 500 times. The accuracy statistics are provided as average over all the iterations. The field AGB (*y*) is fitted to TomoSAR estimated variables [*x*] using linear or nonlinear minimization of the cost function *J* to estimate the model parameters [*a*]. The cost function, *J*, is given by

**Table 2:** Summary of AGB models using different TomoSAR variables.

Model notation	Quantity
Backscattered power at the 30 m layer-based AGB models	
*P1–P15*	It uses single-polarization ${P}^{30}$
*P16–P34*	It uses multi-polarization ${P}^{30}$
Forest height-based AGB models	
*H1–H15*	It uses TFH
Backscattered power at the 30 m layer and Forest height integrated AGB models	
*C1–C9*	It uses single-polarization ${P}^{30}$ and TFH
*C10–C18*	It uses multi-polarization ${P}^{30}$ and TFH
TomoSAR metric-based AGB models. It is the same as Backscattered power at the 30 m layer-based and backscattered power at the 30 m layer and forest height integrated AGB models, except that backscattered power at the 30 m layer is replaced with TomoSAR metric	
*QP1–QP15*	It uses single-polarization Q
*QP16–QP28*	It uses multi-polarization Q
*QC1–QC9*	It uses single-polarization Q and TFH
*QC10–QC18*	It uses multi-polarization Q and TFH

3 $$J= {\underset{[a]}{\mathit{min}}\Vert F\left(\left[a\right],\left[x\right]\right)-y\Vert }_{2}^{2}=\underset{[a]}{\mathit{min}}\sum_{n}{\left(F\left(\left[a\right],\left[x\right]\right)-y\right)}^{2}$$

Once the parameters of AGB models [*a*] are estimated, the corresponding model is calibrated over the entire SAR scene to map the estimated biomass.

4 $${\widehat{y}}_{SAR}=F\left(\left[\widehat{a}\right],\left[{x}_{SAR}\right]\right)$$

Here, ${\widehat{y}}_{\mathrm{SAR}}$ is estimated AGB over the area covered by the SAR image, *F* is the selected AGB model, $\left[\widehat{a}\right]$ is AGB model parameter and $\left[{x}_{\mathrm{SAR}}\right]$ are the TomoSAR variables. The correlation of variables to AGB is assessed using the Pearson correlation coefficient (*r*_*p*_). Further, the quantification of estimation accuracy was performed using the statistical measures; root-mean-square error (*RMSE*) and coefficient of determination (*R*^*2*^).

## Results

Similar to the methodology, the results are TomoSAR processing and tomographic FH are discussed in detail in^47^. In this section, we present the summary for the completeness of this work. No or negligible temporal decorrelation was assumed as the TomoSAR data set was collected within two hours. The phase calibration was performed, and the covariance matrix was estimated using a window size of 9 × 9 (9.0 × 11.205 m) in the range-azimuth direction. Then, the three TomoSAR reconstruction algorithms (BP, Capon, and MUSIC) were applied to generate a multi-layer stack. The generated multi-layer stack represents the backscattered power at a fixed height (say 0, 5, 10…50 m) from the terrain surface. The reflectivity profile and tomographic FH estimates agreed well with Lidar CHM values^47^. With the multi-layer backscattered power values, tomographic FH, and TomoSAR power metric, we performed calibration/validation of AGB models at a 4-ha resolution in further sections.

### AGB estimation from backscattered power at the 30 m layer

**Figure 3:**
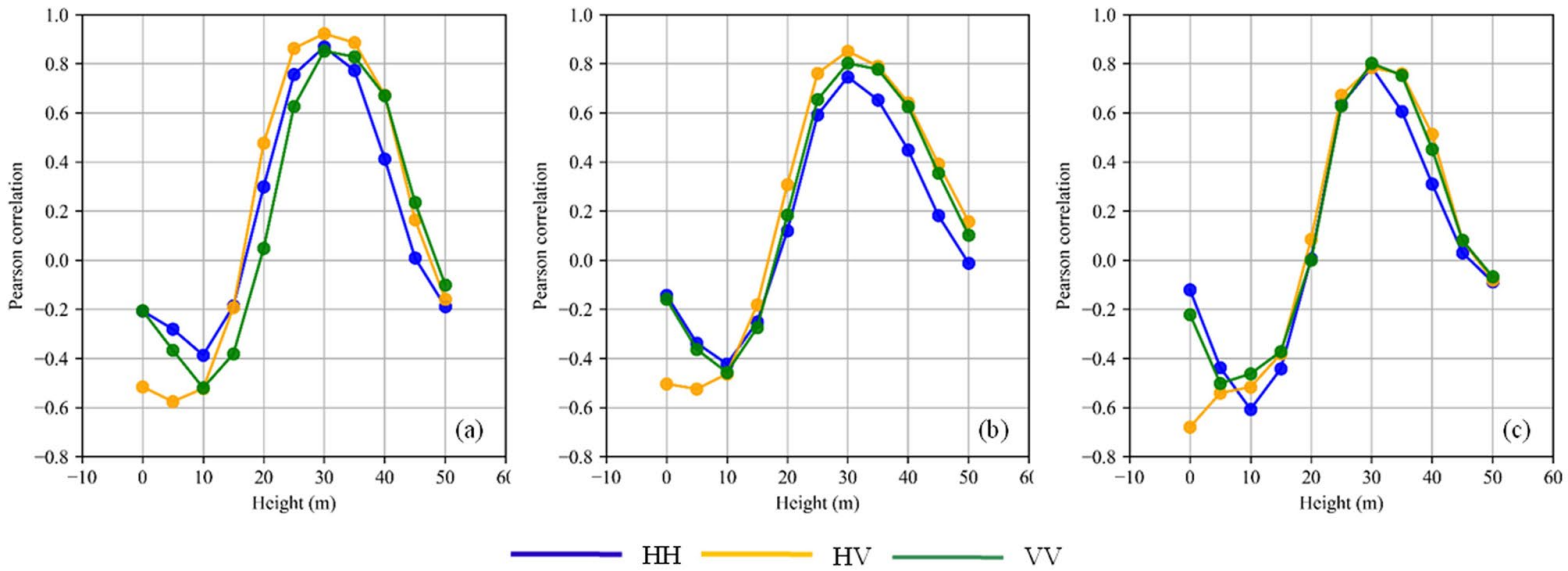
The Pearson correlation of TomoSAR backscattered power associated with different heights reference AGB for different polarizations at *4* ha resolution (**a**) BP, (**b**) Capon, and (**c**) MUSIC*.* Blue, orange, and green represent HH, HV, and VV polarizations.

Firstly, we use the linear regression approach to perform the correlation analysis between the multi-layer stacks and AGB layers. The purpose was two-fold (a) to assess the correlation behavior of the backscattered power of Capon and MUSIC in comparison to the BP multi-layer stack and (b) to assess the impact of different TomoSAR compensation^47^ on AGB estimation. The following can be observed in Fig. 3. (a) BP and MUSIC have the best and worst correlation, respectively. (b) Layers below 15 m had a negative correlation, whereas layers between 25 and 35 m had a strong positive correlation. (c) The maximum correlation was found at the 30 m layer for both the Capon (${r}_{p}$ = 0.85) and MUSIC (${r}_{p}$ = 0.80) estimators, similar to BP (0.92) estimator. (d) The correlation of HV (${r}_{p}^{BP}$=− 0.51, ${r}_{p}^{Capon}$= − 0.50, ${r}_{p}^{MUSIC}$= − 0.68) at ground level is quite different from HH (${r}_{p}^{BP}$=− 0.21, ${r}_{p}^{Capon}$= − 0.14, ${r}_{p}^{MUSIC}$= − 0.12) and VV (${r}_{p}^{BP}$=− 0.21, ${r}_{p}^{Capon}$= − 0.16, ${r}_{p}^{MUSIC}$= − 0.22) polarization.

Analyzing the correlation of different TomoSAR compensations revealed that slope compensation backscattered (TGS) values had the highest correlation values (Supplementary Table S3). Hence, we will use the backscattered power at the 30 m layer, the tomographic FH, and the TomoSAR backscatter power metric (Q) estimated from slope-compensated multi-stack for AGB retrieval. Apart from linear regression (*P1–P3*), we also examined pure-quadratic (*P4–P6*), exponential (*P7–P9*), power series (*P10–P12*), and sigmoid (*P13–P15*) models to understand the relation between the backscattered power at the 30 m layer and AGB. It was observed that for all models employing the backscattered power at the 30 m layer estimated from BP, AGB values below 350 Mg ha^−1^ indicate a good fit, but values above 350 Mg ha^-1^ indicate greater dispersion. However, higher dispersion is observed for both the Capon and MUSIC estimators, particularly the MUSIC estimator (Supplementary Fig. S2). Comparing these models, the linear and pure-quadratic models performed consistently the best for all model-polarization combinations (Supplementary Table S4). The best result is obtained using the BP estimator at HV, with the lowest RMSE and *R*^*2*^ values of 17 Mg ha^−1^ and 0.86, respectively, for the pure-quadratic model. The pure-quadratic model also consistently performed with Capon (RMSE = 21.93 Mg ha^−1^, *R*^*2*^ = 0.76) and MUSIC (RMSE = 25.35 Mg ha^−1^, *R*^*2*^ = 0.68) ${P}^{30}$, and hence will be considered the baseline models for the rest of the analysis. The exponential model performed the worst. It can be seen from higher *RMSE* and *R*^*2*^ values that the exponential model tends to overfit the values.

We adopted linear and pure-quadratic models to combine multiple polarization data based on the above results. In general, a degradation or minor improvement in accuracy was observed for BP and MUSIC, while for Capon, significant improvement was noticed compared to their baseline models (Supplementary Table S5). In addition, non-linear polarization states were synthesized to assess how the addition of synthesized polarization information impacts the performance of multi-polarization-based AGB models. The combination of the backscattered power at the 30 m layer in PiH, HH, and HV polarization using BP improved the accuracy of the estimator for both linear (RMSE = 14.31 Mg ha^−1^, *R*^*2*^ = 0.9) and pure-quadratic (RMSE = 14.73 Mg ha^−1^, *R*^*2*^ = 0.89) models. However, no such behavior was observed for Capon and MUSIC estimators (Supplementary Table S6).

### AGB estimation from tomographic FH

The tomographic FH was estimated at a 4-ha resolution with an RMSE of less than 10% using the BP, Capon, and MUSIC estimators^47^. Now, similar to the backscattered power at the 30 m layer, we studied the linear (*H1–H3*), pure-quadratic (*H3–H6*), exponential (*H7–H9*), power series (*H10–H12*), and sigmoid (*H13–H15*) models to comprehend the relation between tomographic FH and AGB (Supplementary Table S7). None of these models, however, outperformed the base models. Interestingly, while tomographic FH estimation based on BP slightly underperformed compared to MUSIC and Capon, the tomographic FH-based AGB estimations using BP performed better than MUSIC and Capon. Among the tomographic FH-based models, the BP FTH at HV polarization was the most accurate (RMSE = 23 Mg ha^−1^, *R*^*2*^ = 0.73). Compared to base models, the tomographic FH-based models displayed an average RMSE increment of ~ 7–15 Mg ha^−1^, depending on polarization-model combinations.

### AGB estimation from Integrated the backscattered power at the 30 m layer and tomographic FH

The integration of the backscattered power at the 30 m layer and tomographic FH (*C1–C18*) increased the accuracy of the AGB estimates. The relative RMSE improved from ~ (5–12)%—and ~ (7–11)% for the backscattered power at the 30 m layer- and tomographic FH-based AGB models, respectively, to ~ (4–7) % for combined models. Notably, the exponential (*C7–C9*) AGB models integrating the backscattered power at the 30 m layer and tomographic FH displayed significant improvement compared to the exponential models using backscattered power at the 30 m layer (*P7–P9*). The RMSE improved from ~ 39 to ~ 42 Mg ha^−1^ to ~ 15 to ~ 23 Mg ha^−1^ polarization-model combinations. Compared to the base model, the best accuracy was achieved by combining the backscattered power at the 30 m layer and tomographic FH at HV using BP estimator and pure-quadratic AGB model (RMSE 16.60 to 12.23 Mg ha^−1^).

### AGB estimation from TomoSAR power metric

Apart from backscattered power at the 30 m layer and tomographic FH, we explored using the derived TomoSAR power metric to estimate AGB. We restrict the analysis to the TomoSAR power metric computed from multi-stack using the BP estimator for simplicity. It was observed that apart from *Q4,* none of the other TomoSAR power metric metrics showed a considerable relationship with reference AGB (Supplementary Fig. S3). First, we use the TomoSAR power metric independently to estimate the AGB (*QP1–QP24*). These models are similar to that of ${P}^{30}$-based models, except that the backscattered power at the 30 m layer is replaced with the TomoSAR power metric metrics. The *Q4* metric could achieve the best accuracy (RMSE = 16.28 Mg ha^−1^, *R*^*2*^ = 0.87) at HV polarization using a pure-quadratic model (*QP*5), which is a minor improvement compared to the base model. Also, combining the V-receive polarization improved the accuracy further to RMSE = 15.83 Mg ha^−1^ and *R*^*2*^ = 0.88. Adding tomographic FH information and the TomoSAR power metric (QC1-QC18) significantly improved all TomoSAR power metrics other than Q4, where minimal or negligible improvement is observed. The best observation was still observed for the *Q4* metric (RMSE = 14.49 Mg ha^−1^, *R*^*2*^ = 0.89). However, this accuracy was lower than the best possible accuracy achieved by integrating the backscattered power at the 30 m layer and tomographic FH-based model but at a higher computational cost.

### Comparison of TomoSAR and LIDAR FH and AGB

Currently, of all RS techniques, the best estimates of FH are obtained using LiDAR. However, the global coverage is limited by data sampling. Hence, Lidar-derived FH is expected to produce reliable AGB that can be used as a proxy for in situ measurements of forest structure and biomass. Here, we perform the comparison of (a) tomographic and LiDAR FH, (b) AGB estimates derived from LiDAR FH, tomographic FH, the backscattered power at the 30 m layer from BP estimator at HV polarization using the *P2* model, and (c) AGB modeled using ground and Lidar FH-derived AGB data. The tomographic FH used in this section is estimated at HV polarization. To derive AGB from LiDAR and tomographic FH, we have used the power law model adopted by^54^ and given by

5 $$\widehat{W}=a{H}_{x}^{b}$$

**Figure 4:**
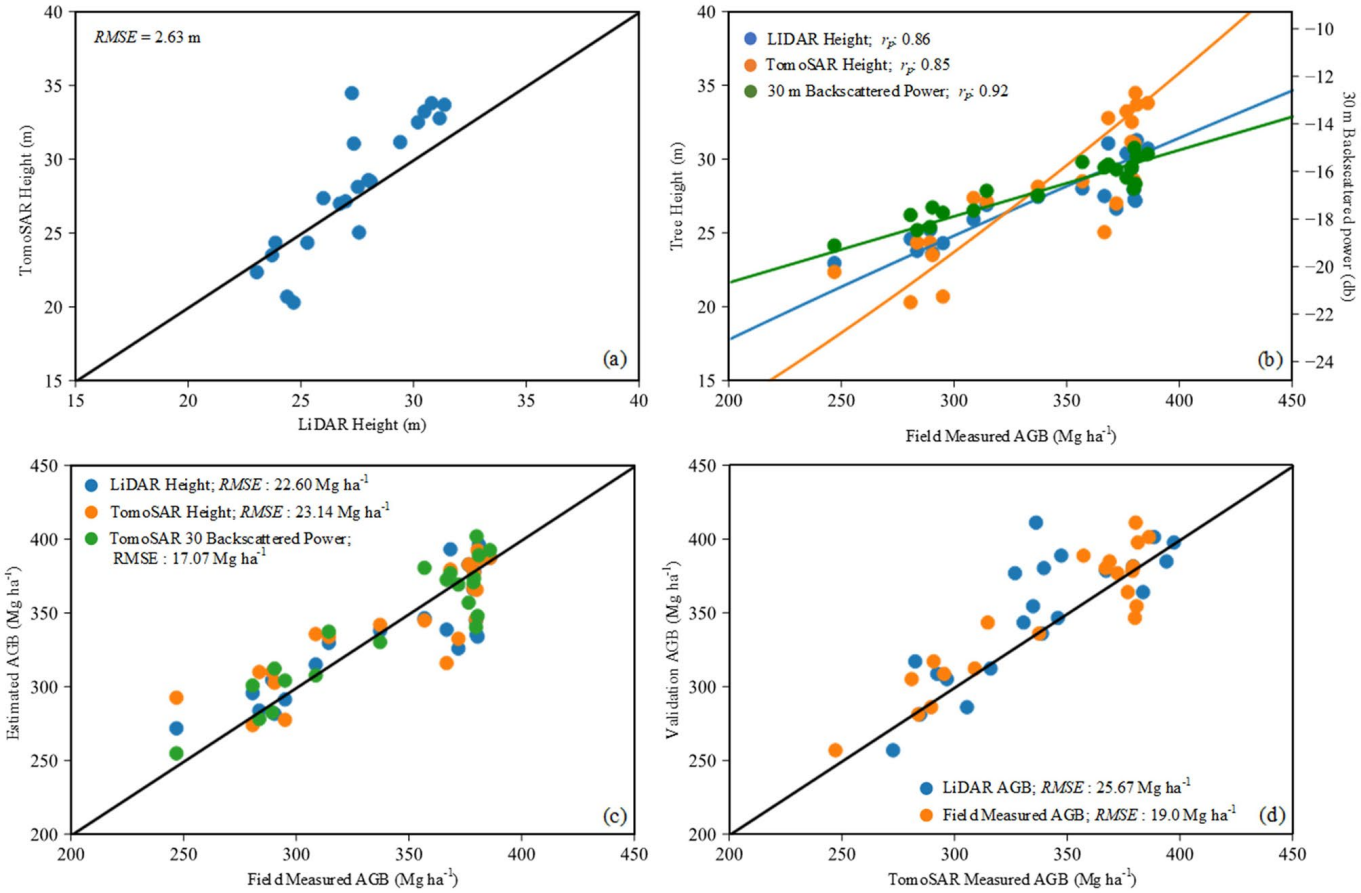
The TomoSAR AGB validation using field measured and LiDAR AGB.

Here*,* ${H}_{x}$ is tomographic FH or LiDAR FH. The comparison of height estimates at the 4-ha plot scale shows reasonable agreement across the entire range, with tomographic FH showing slight overestimation for the plots with the tallest average height (Fig. 4a). When the correlation between the ground AGB and the Lidar FH, tomographic FH, and the backscattered power at the 30 m layer from the BP estimator at HV polarisation is examined (Fig. 4b), it can be seen that, although these parameters show reasonable correlation, both FH exhibited increased dispersion. While the LiDAR FH dispersion is greater for AGB > 350 Mg ha^−1^, the tomographic FH dispersion is greater for most AGB ranges. Further, comparing the AGB estimates, the backscattered power at the 30 m layer showed the best correlation (0.92) with much lesser dispersion and estimated AGB with the least RMSE (17.07 Mg ha^−1^) followed by LiDAR AGB estimates (*r*_*p*_ = 0.86; RMSE = 22.6 Mg ha^−1^) (Fig. 4c). Finally, we calibrated the *P1* AGB model using Lidar FH-derived AGB estimates and validated it against both LiDAR FH-derived and ground AGB to verify the reliability of utilizing LiDAR FH-derived AGB to calibrate and validate TomoSAR AGB models. AGB estimates calibrated using LiDAR FH-derived AGB are found to be overestimated when compared to LiDAR FH-derived AGB estimates, but comparing it to ground AGB, gives a much better RMSE of 19.0 Mg ha^−1^ (Fig. 4d).

## Discussion

This study demonstrates that P-band TomoSAR techniques can accurately retrieve AGB tropical forests even over varying AGB and terrain conditions. The analysis shows that a less than ten percent relative error can be achieved using the combination of multiple variables derived from the TomoSAR over the Paracou test site. We also highlight the importance of selecting a robust processing chain to obtain reliable and accurate AGB estimates. Finally, we demonstrate that LiDAR FH and AGB can be used as a proxy to improve the AGB estimates from TomoSAR variables. These results boost the possibility of using GEDI and BIOMASS data in combination to provide precise wall-to-wall AGB mapping.

Firstly, we analyzed the correlation of AGB with different layers of a multi-layer stack using BP, Capon, and MUSIC estimators. The ground-level layer showed distinct characteristics in the cross-polarized channel compared to co-polarized channels. This behavior can be attributed to lower ground contributions in HV than in co-polarized channels^70^. The sidelobe from the canopy and ground signals contribute to the intensity of ground-level tomography. When the contribution of the ground is reduced, the contribution of the canopy’s sidelobe becomes dominant. As a result, there is a significant negative correlation observed. This behavior is observed for all estimators. Also, analyzing the impact of different backscatter compensation approaches on AGB estimates, we found that the slope-compensated tomographic power provides the best correlation with AGB compared to other compensations for the BP estimator. Although, the volume-compensated tomographic also displayed a similar level of accuracy over the Paracou test site, where vertical resolution is around 20 m and does not show significant variation over the plot. Hence, to assess the efficiency of this compensation, it has to be validated over plots with variable vertical resolution and will be treated as a separate problem and not considered in this paper. Another important observation was that even though the slope compensated tomographic power for BP significantly improved its correlation with AGB, it resulted in a degradation of correlation for Capon and MUSIC estimators.

**Figure 5:**
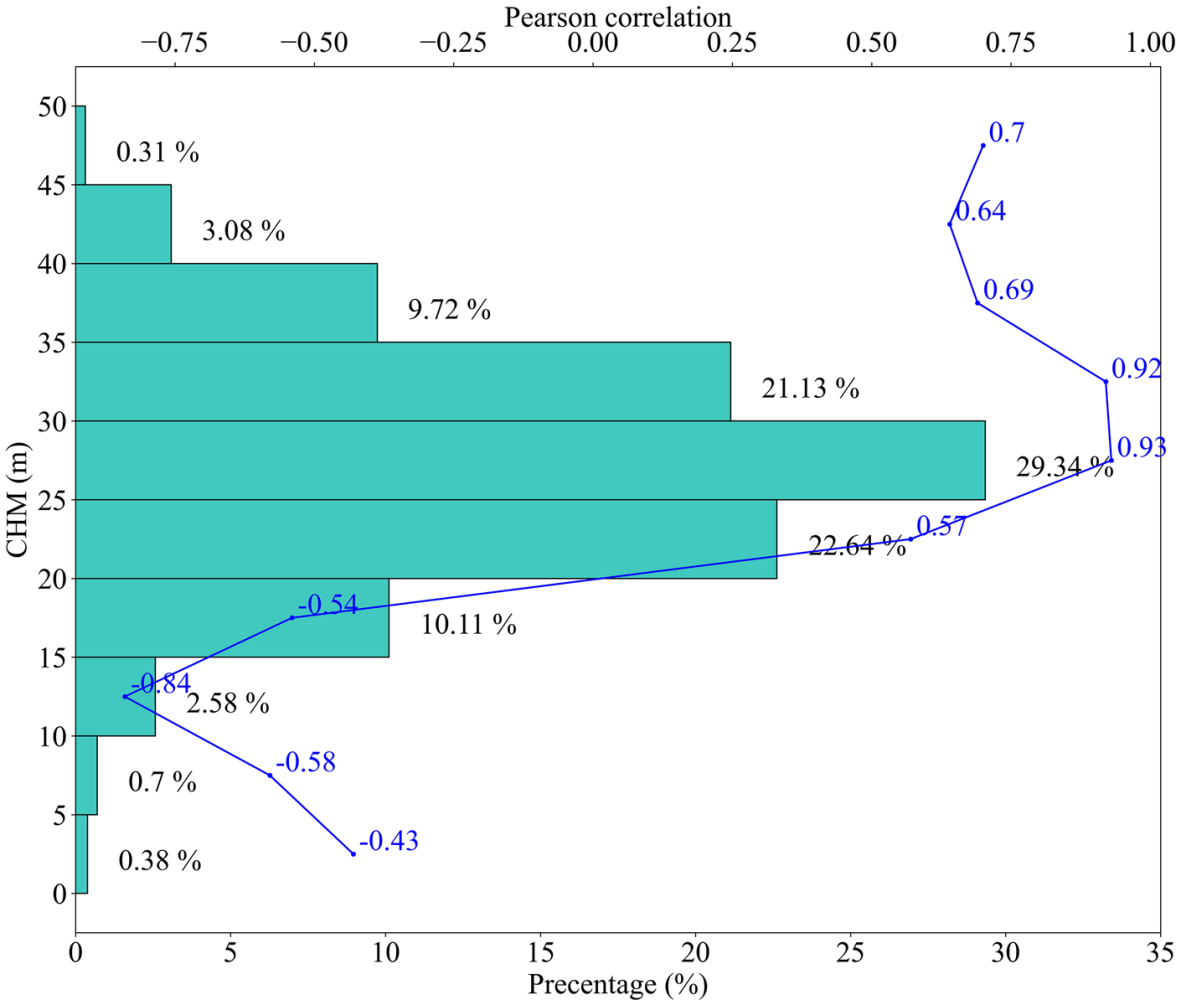
Percentage histogram of LiDAR CHM within the plot region. The correlation of AGB with the associated height bin is also indicated using a line diagram (blue).

Comparing all estimator-polarization combinations, the best performance was achieved using the backscattered power at the 30 m layer at HV polarization using the BP estimator. With the vertical resolution of the 20 m over the Paracou dataset, the backscattered power at the 30 m layer represents the backscatter from scatterers between 20 and 40 m. Previous ecological studies reinforce these results^42,43^. Also, we compared the distribution of LiDAR CHM over the plots and observed that around 83 percent of CHM falls within the 20–40 m range (Fig. 5). Further, we assessed the correlation pattern of the mean of Lidar CHM over the bins (0–5, 5–10, 10–15… 45–50 m) with AGB. It can be observed that the best correlation was achieved for bin 25–30 m, followed by 30–35 m. However, for Capon and MUSIC estimators, the reduction in accuracy can be attributed to (a) improved vertical resolution: theoretically, the amount of AGB content with finer resolution cells should be ideally less. However, detailed work on the vertical resolution of the Capon and MUSIC estimator and the content within the 30 m resolution cell has to be investigated further and is out of the scope of this paper. (b) Loss in radiometric quality: it is well established that Capon and MUSIC estimators are more accurate for point scatters and not optimized for volume scatterers, leading to degraded power estimates^71^.

The result of the top-performing models (Supplementary Table S4-S10) is summarized in Tables 3 and 4. The linear and pure quadratic models performed best with higher *R*^*2*^ and lower RMSE values compared to single polarimetric-based models. The larger dispersion is seen for AGB values greater than 350 Mg ha^−1^ and results in an underestimation of AGB estimates. However, most of the multi-polarimetric-based models showed lesser dispersion with improved accuracy. The model combining HH and HV and synthesized PiH polarization showed the best results. This gives an implication that a dual-polarization system can be adopted for the TomoSAR mission, as these combinations can be derived using a dual polarimetric system (HH + HV or VV + VH, and a third one can be derived using a combination of these), and it is possible to achieve improved accuracy. Integrating the backscattered power at the 30 m layer and tomographic FH to estimate AGB indicate improved retrievals compared to using them independently. This improvement is much more significant in combined models than in backscatter or height models for single polarization. This confirms that combined usage of TomoSAR variables helps counterbalance the perturbations that affect them. These models provide the best overall performance for AGB estimates concerning the study area. This is confirmed by the coefficient of determination values, which is consistently high for all the models.

**Table 3:** Summary of ${P}^{30}$, TFH, integrated ${P}^{30}$ at different polarization and integrated ${P}^{30}$ and TFH AGB models.

Model	AGB model	BP		Capon		MUSIC	
		*RMSE*	*r*^*2*^	*RMSE*	*r*^*2*^	*RMSE*	*r*^*2*^
*P2*	$y={a}_{0}+{a}_{1}{P}_{HV}$	17.07	0.85	23.32	0.72	27.41	0.62
*P5*	$y={a}_{0}+{a}_{1}{P}_{HV}+{a}_{2}{P}_{HV}^{2}$	16.60	0.86	21.93	0.76	25.67	0.67
*P19*	$y={a}_{0}+{{a}_{1}{P}_{HV}+{a}_{2}{P}_{HV}^{2}+a}_{3}{P}_{VV}+{a}_{4}{P}_{VV}^{2}$	16.14	0.87	22.84	0.75	26.40	0.66
*P23*	$y={a}_{0}+{{a}_{1}{P}_{HH}+{a}_{2}{P}_{HH}^{2}+a}_{3}{P}_{HV}+{a}_{4}{P}_{HV}^{2}+{a}_{5}{P}_{VV}+{a}_{6}{P}_{VV}^{2}$	16.27	0.86	19.69	0.82	26.64	0.64
*P25*	$y={a}_{0}+{{a}_{1}{P}_{HH}+a}_{2}{P}_{HV}+{a}_{3}{P}_{PiH}$	14.31	0.90	19.74	0.80	26.91	0.63
*P30*	$y={a}_{0}{{+a}_{1}{P}_{HH}+{a}_{2}{P}_{HH}^{2}+a}_{3}{P}_{HV}+{a}_{4}{P}_{HV}^{2}+{a}_{5}{P}_{PiH}+{a}_{6}{P}_{PiH}^{2}$	14.73	0.89	19.74	0.80	26.91	0.63
*H5*	$y={a}_{0}+{a}_{1}{H}_{HV}+{a}_{2}{H}_{HV}^{2}$	23.06	0.73	26.48	0.64	27.31	0.63
*C5*	$y={a}_{0}+{{a}_{1}{P}_{HV}+{a}_{2}{P}_{HV}^{2}+a}_{3}{H}_{HV}+{a}_{4}{H}_{HV}^{2}$	12.23	0.92	17.87	0.84	22.13	0.75
*C16*	$y={a}_{0}+{{a}_{1}{P}_{HH}+a}_{2}{P}_{HV}+{a}_{3}{P}_{VV}+{{a}_{4}H}_{VV}$	13.59	0.91	19.42	0.81	21.76	0.76

**Table 4:** Summary of TomoSAR power metric (Q) and integrated Q & TFH AGB models.

Model	AGB model	Q1		Q2		Q3		Q4		Q5	
		*RMSE*	*r*^*2*^	*RMSE*	*r*^*2*^	*RMSE*	*r*^*2*^	*RMSE*	*r*^*2*^	*RMSE*	*r*^*2*^
*QP5*	$y={a}_{0}+{a}_{1}{{Q}_{n}}_{HV}+{a}_{2}{{Q}_{n}}_{HV}^{2}$	44.01	0.02	42.82	0.08	42.91	0.06	16.28	0.87	44.32	0.05
*QP19*	$y={a}_{0}+{{a}_{1}{{Q}_{n}}_{HV}+{a}_{2}{{Q}_{n}}_{HV}^{2}+a}_{3}{{Q}_{n}}_{VV}+{a}_{4}{{Q}_{n}}_{VV}^{2}$	44.98	0.05	45.63	0.09	40.54	0.21	15.83	0.88	46.35	0.03
*QC5*	$y={a}_{0}+{{a}_{1}{{Q}_{n}}_{HV}+{a}_{2}{{Q}_{n}}_{HV}^{2}+a}_{3}{H}_{HV}+{a}_{4}{H}_{HV}^{2}$	15.75	0.88	29.77	0.76	19.97	0.80	14.49	0.89	19.95	0.84

We also investigated the capability of TomoSAR power metrics to estimate AGB because the fixed height layer analysis does not geometrically and physically justify the canopy height range of global forest biomes and the associated AGB. Among the five metrics, the Q4 metric performed exceptionally well. Similar to the backscattered power at 30 m, Q4 integrates the contribution primarily from the intermediate TomoSAR cells, which contain a high amount of AGB components, resulting in a stronger correlation with AGB values compared to other metrics^40,41^. proposed a metric similar to Q4 (but integrating between the fixed height range of 10–30 m) and observed that it correlated better with AGB than conventional SAR backscatter values. However, the effect of vertical resolution on Q4 will play a vital role in deciding the influence of ground contribution on this metric. Further, studies must be incorporated to understand the impact of different vertical resolutions and their corresponding ground contribution effect on integrated power metrics. In contrast, the possible reason for the degrading performance of other metrics can be explained as follows: With an average tree height of around 27 m across the plots, the Q2 metric should yield a backscattered power of about 20–15 m. As a result, the low correlation of Q2 with AGB can be attributed to power loss caused by high tree penetration as it propagates toward the ground. Previous research^25,28,39^ has also discussed the site’s weaker correlation for layers below 25 m. Similarly, the Q3 metric should yield a backscattered power of between 35 and 50 m. With the maximum average forest height of 45 m over the plots, the loss in the correlation of AGB to Q3 metric can be primarily attributed to the backscatter contribution from noise regions in the vertical distribution model, which may not be associated with any physical component of forests. Also, the phase center for the study site lies below 15 m^37^. The weaker correlation with the Q5 metric can be due to the dominance of effect due to slope and attenuation, which is more prominent in lower layers.

Now, compared to the backscattered power at the 30 m layer, LiDAR and tomographic FH generated less accurate AGB estimates. One of the possible reasons for this may be because of the limitation of LiDAR to reach the ground over the dense, multi-storied forest, leading to errors in FH estimates that may be reflected in AGB. A recent study using the GEDI data has reported higher uncertainty in AGB over multi-layered forests^50^. This suggests that height alone cannot represent AGB in tropical conditions, regardless of the approaches utilized, and that a multivariable model is required to quantify AGB estimates more precisely. Combining the backscattered power at the 30 m layer with FH improved the accuracy, demonstrating that it is possible to counteract the accuracy loss due to the effect of the ground contribution that contaminates the backscattered signal from the forest layers and achieve accurate AGB estimates.

## Conclusion

The analysis and results presented in this paper evaluate the ability of different variables derived from TomoSAR measurements to estimate AGB in the framework of ESA’s P-Band SAR mission BIOMASS. The analysis is performed based on the data collected over the Paracou test site during the TropiSAR airborne campaigns in 2009. The results suggest the following: (1) irrespective of the reconstruction algorithm used, the backscattered power at 30 m height from ground show the highest correlation with AGB. The backscattered power at 30 m height estimated using BP provided the best results of the three reconstruction algorithms. Comparing the different polarization HV polarization demonstrated the higher accuracy across different reconstruction algorithms. Further, in this study, we observed that slope compensated backscattered power perform better than the volume compensated backscattered power. However, this result has to be reaffirmed by experimenting with datasets at different vertical resolution. (2) Compared with tomographic FH, the backscattered power at the 30 m layer estimated the AGB values more accurate, and their integration further improved the AGB estimation, counteracting the limitations in each. Also, the use of integrated power metric (Q4) shows promising results and can serve as an alternative for the backscattered power at the 30 m layer. However, further studies should be carried out to understand the effect of vertical resolution on the overall accuracy in different conditions. (3) Comparison of FH estimation from LiDAR and TomoSAR showed better correlation. However, the AGB estimates using both FH products, underperformed compared to the backscattered power at the 30 m layer, indicating tree height is not alone sufficient to represent AGB. However, to address the global lack of field data, calibrating the AGB models with LiDAR-derived AGB can still provide a high level of AGB accuracy across the forest landscape. (4) It is well known that the best height estimates are estimated by LiDAR data, while we have seen that the best tomographic variable that provide the best accuracy for AGB estimates is the backscattered power at the 30 m layer. Therefore, the combination of these two variables could be effectively used for regional and global AGB mapping. To date, the spaceborne TomoSAR and LiDAR data are not available over a common operational period. However, with overlapping operational period of BIOMASS and GEDI sensor, the presented results demonstrate the capability of using combined AGB models to improve our global AGB estimates significantly and assist global wall-to-wall AGB maps.

## Supplementary Information

Supplementary Information 1.

Supplementary Information 2.

Supplementary Information 3.

Supplementary Information 4.

## Data Availability

The data used in this study are available upon reasonable request from the ESA under the EO campaign dataset. https://doi.org/10.5270/esa-1rtogy6.
