# Supplementary figures and images for: Mapping tropical forest aboveground biomass using airborne SAR tomography
Source: Sci Rep. 2023 Apr 17;13:6233. doi: 10.1038/s41598-023-33311-y (PMC10110524; doi:10.1038/s41598-023-33311-y)

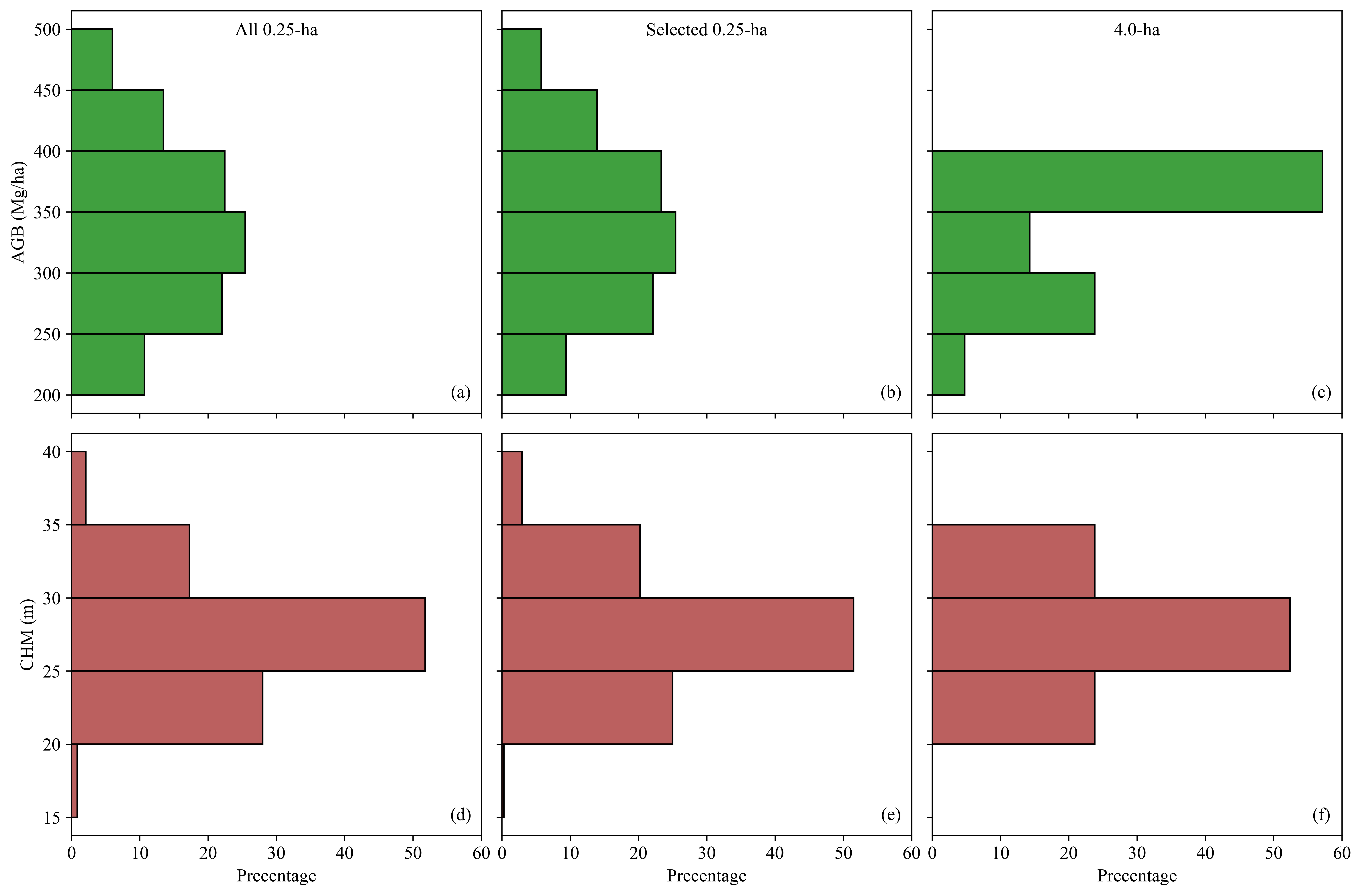

Supplement: Supplementary file 1 — Supplementary Information 1. [file 41598_2023_33311_MOESM1_ESM.jpg]

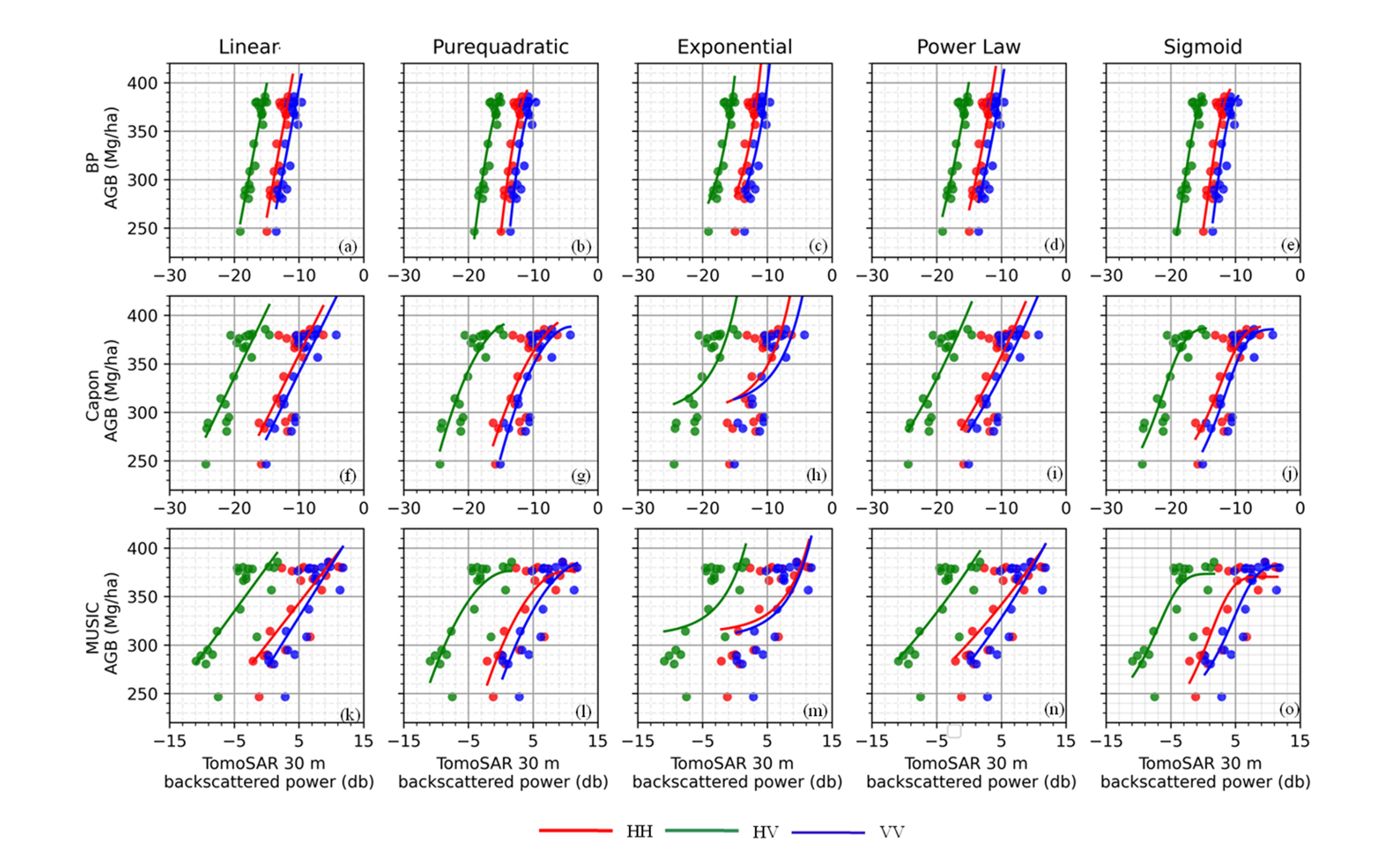

Supplement: Supplementary file 2 — Supplementary Information 2. [file 41598_2023_33311_MOESM2_ESM.jpg]

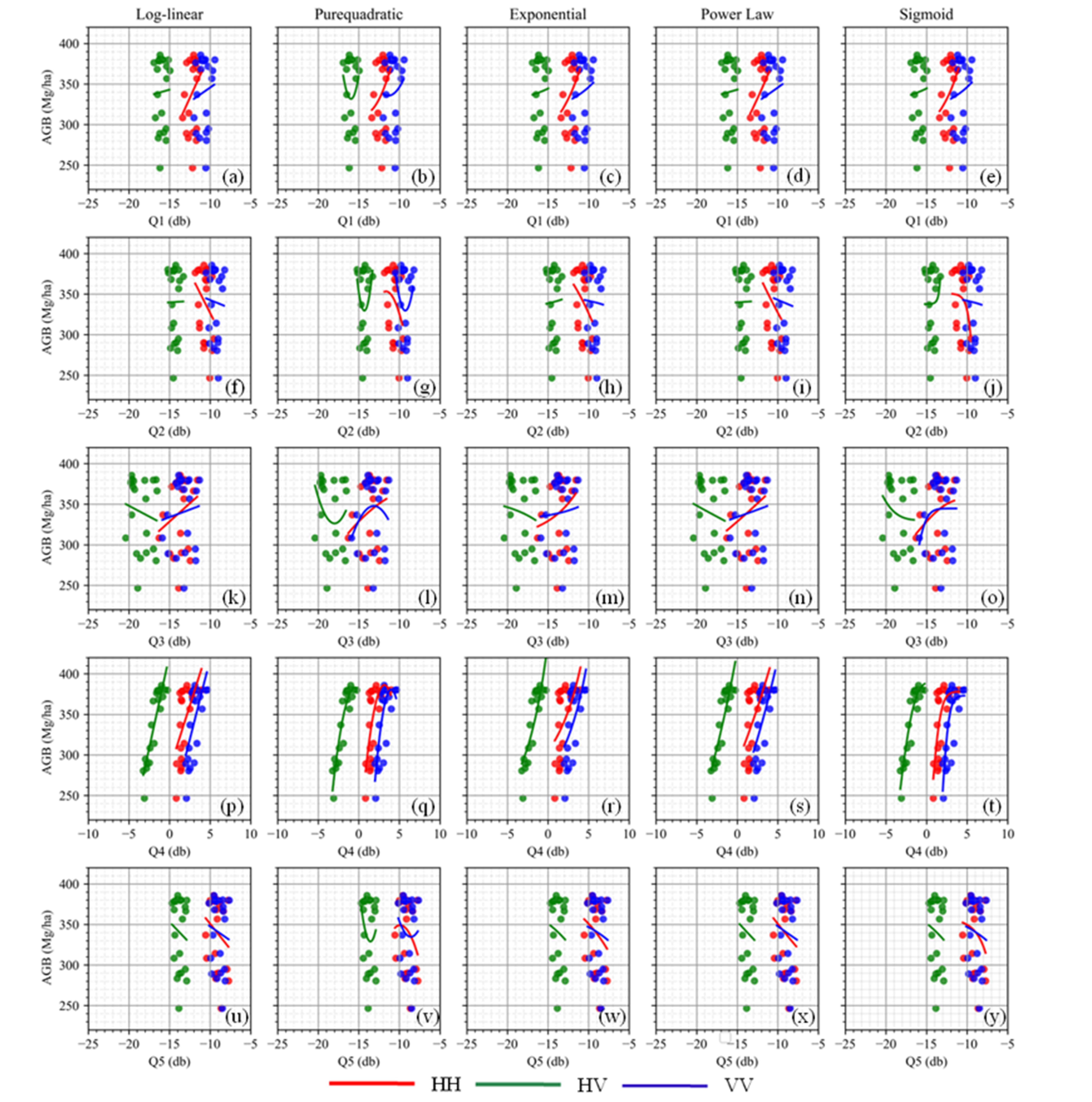

Supplement: Supplementary file 3 — Supplementary Information 3. [file 41598_2023_33311_MOESM3_ESM.jpg]
