# Supplementary material for: Mapping tropical forest aboveground biomass using airborne SAR tomography
Source: Sci Rep. 2023 Apr 17;13:6233. doi: 10.1038/s41598-023-33311-y (PMC10110524; doi:10.1038/s41598-023-33311-y)
Supplement: Supplementary file 4 — Supplementary Information 4. [file 41598_2023_33311_MOESM4_ESM.docx]

Mapping Tropical Forest Above-ground Biomass using Airborne SAR Tomography

Naveen Ramachandran^1, *^, Sassan Saatchi^2^, Stefano Tebaldini^3^, Mauro Mariotti d’Alessandro^3^, and Onkar Dikshit^1^

^1^ Department of Civil Engineering, Indian Institute of Technology Kanpur, Kanpur 208016, India
^2^ National Aeronautics and Space Administration (NASA), Jet Propulsion Laboratory (JPL), California Institute of Technology, Pasadena, CA 91125, USA

^3^ Dipartimento di Elettronica, Informazione e Bioingegneria, Politecnico di Milano, 20133 Milan, Italy.
^*^ naveenr342@gmail.com

**Supplementary material**

**Supplementary Method**

**Supplementary Method S1.** The TomoSAR reflectivity profiles are reconstructed for the BP, Capon, and MUSIC estimators as

| $P_{BP}\left( x,r, v \right)=\frac{1}{K}\left\vert\sum_{k=1}^{K} \left\langle{S_{k}\left( x,r \right)e}^{\left( -j{K_{z}}_{k}v \right)} \right\rangle\right\vert^{2}$  $P_{CB}\left( x,r, v \right)= \frac{1}{a^{H}\left( v \right).\hat{R}^{-1}.a(v)}$  $P_{MU}\left( x,r,v \right)= \frac{1}{a^{H}\left( v \right).W_{n}.W_{n}^{H}.a(v)}$ | (1) |
| --- | --- |

Here,$P_{\mathrm{BP}}, P_{CB}$are the average scene complex reflectivity and $P_{MU}$is the average scene complex pseudo-reflectivity within the azimuth-slant range resolution at height layer $z$from the ground level using estimator $m$ ($m$ can be BP or CB or MUSIC). $\left\langle\cdot\right\rangle$ is the averaging performed in the range-azimuth direction. $S_{k}\left( x,r \right)$denotes the complex-valued pixel located at azimuth, slant range position $\left( x,r \right)$ from the k^th^ sensor,$v$ is the cross-range coordinate perpendicular to the platform motion and direction of the radar signal. Further, $\hat{R}$is the estimated covariance matrix, and $a(z)$ denotes the steering vector given by equations (2b) and (3) and$W_{n}$ represents the eigenvectors of the noise subspace.

| $S_{MB}=\left[ \begin{matrix} S_{1}\left( x,r \right) & S_{2}\left( x,r \right) & \begin{matrix} \ldots& S_{K}\left( x,r \right) \end{matrix} \end{matrix} \right]^{T}$ | (2a) |
| --- | --- |
| $\left\langle\hat{R} \right\rangle_{K\times K}= \left\langle S_{MB}S_{MB}^{H} \right\rangle$ | (2b) |
| $a\left( z \right)=\left[ \begin{matrix} 1, & exp(jK_{z_{2}}z), & \begin{matrix} expjK_{z_{3}}z), & \ldots, & exp(jK_{z_{K}}z) \end{matrix} \end{matrix} \right]^{T}$ | (3) |

Here, $K_{z}$ represents the vertical wave number and can be related to the Height of Ambiguity (HoA) between the interferometric pairs.

| $K_{z}=\frac{2\pi}{HoA}=\frac{4\pi}{\lambda r_{0}}b_{n}$ | (4) |
| --- | --- |

The geometrical resolution ($\Delta v$) in the cross-range can be expressed as in [1] and TomoSAR grids are sampled at 1 m. The resolution in the vertical direction can be expressed by the exploitation of a simple geometrical relation $z=v.\sin\theta$, where $\theta$ is the elevation angle.

| $\Delta v\approx\frac{\lambda r_{0}}{2.L_{tomo}}\approx\frac{2\pi}{Max\left( K_{z} \right)-Min(K_{z})}\sin\theta$ | (5) |
| --- | --- |

**Supplementary Method S2.** The forest tree height is estimated from the TomoSAR reflectivity cube. This approach's key idea is to investigate the shape formed by the vertical distribution of reflectivity ($P_{m}$ (z, g, r)) for each azimuth-ground range index at height layer $z$from the ground level using estimator $m$ ($m$ can be BP or CB or MUSIC). Here, we assume that the shape of the reflectivity function along the vertical may be divided into three zones: backscattering from the canopy layer, especially the phase center ($H_{c}$), power loss region (*K*), and noise region. The height is retrieved by varying the power loss value from the phase center located along the upper envelope in the vertical direction. This is given by

| $FTH\left( x,g \right)=argmin\left\{ \left\vert P_{m}\left( z^{'},x,g \right)-P_{m}\left( H_{c},x,g \right)-K \right\vert\right\}$ | (6) |
| --- | --- |

Here, $P_{m}\left( H_{c},x,g \right)$ is the backscattered power at $H_{c}$, $P_{m}\left( z^{'},x,g \right)$is the backscattered power at a different elevation ranging from $H_{c}$ to the upper part of the profile, *x* stands for BP, Capon (CB), or MUSIC (MU) estimators. The effective phase center is therefore estimated as:

| $H_{c}\left( x,g \right)=argmax\left\{ P_{m}(z,x,g) \right\}$ | (7) |
| --- | --- |

**Supplementary Tables**

**Table S1:** The ground AGB stats using all 0.25-ha plots, selected 0.25-ha plots for 4-ha plot generation, and 4-ha plots

|  | **All 0.25 ha plots** | | **Selected 0.25 ha plots** | | **Aggregated 4-ha plots** | |
| --- | --- | --- | --- | --- | --- | --- |
|  | **AGB (Mg ha^-1^)** | **Height (m)** | **AGB (Mg ha^-1^)** | **Height (m)** | **AGB (Mg ha^-1^)** | **Height (m)** |
| **Min** | 169.0 | 19.25 | 169.0 | 20.25 | 246.57 | 23.00 |
| **Max** | 618.0 | 36.83 | 618.0 | 36.83 | 385.66 | 31.34 |
| **Mean** | 338.45 | 26.90 | 340.26 | 27.29 | 340.26 | 27.29 |
| **Standard Deviation** | 71.08 | 3.20 | 71.08 | 3.22 | 44.64 | 2.57 |

**Table S2:** The Pearson correlation coefficients between reflectivity and field AGB at different heights using BP, Capon, and MUSIC estimators at 4-ha resolution.

| **Method** | **Pol.** | **Height** | | | | | | | | | | |
| --- | --- | --- | --- | --- | --- | --- | --- | --- | --- | --- | --- | --- |
|  |  | **0** | **5** | **10** | **15** | **20** | **25** | **30** | **35** | **40** | **45** | **50** |
| **BP** | **HH** | -0.21 | -0.28 | -0.39 | -0.19 | 0.30 | 0.76 | 0.87 | 0.77 | 0.41 | 0.01 | -0.19 |
|  | **HV** | -0.52 | -0.58 | -0.52 | -0.19 | 0.48 | 0.86 | 0.92 | 0.89 | 0.67 | 0.16 | -0.16 |
|  | **VV** | -0.21 | -0.37 | -0.52 | -0.38 | 0.05 | 0.63 | 0.85 | 0.83 | 0.67 | 0.24 | -0.10 |
| **Capon** | **HH** | -0.14 | -0.34 | -0.42 | -0.25 | 0.12 | 0.59 | 0.75 | 0.65 | 0.45 | 0.18 | -0.01 |
|  | **HV** | -0.50 | -0.52 | -0.46 | -0.18 | 0.31 | 0.76 | 0.85 | 0.79 | 0.64 | 0.39 | 0.16 |
|  | **VV** | -0.16 | -0.36 | -0.46 | -0.27 | 0.18 | 0.65 | 0.80 | 0.78 | 0.62 | 0.35 | 0.10 |
| **MUSIC** | **HH** | -0.12 | -0.44 | -0.61 | -0.44 | 0.01 | 0.63 | 0.79 | 0.61 | 0.31 | 0.03 | -0.09 |
|  | **HV** | -0.68 | -0.54 | -0.52 | -0.38 | 0.09 | 0.67 | 0.78 | 0.76 | 0.51 | 0.08 | -0.08 |
|  | **VV** | -0.22 | -0.50 | -0.46 | -0.37 | 0.00 | 0.63 | 0.80 | 0.75 | 0.45 | 0.08 | -0.07 |

**Table S3:** The Pearson correlation coefficients between the backscattered power at the 30 m layer with different TomoSAR compensation and filed AGB for HV polarization at 4-ha resolution. Here UC and RC stand for uncorrected and radiometric corrected. TGS and TGV stand for slope and volumetric correction. Refer to [2] for a detailed explanation of RC, TGS, and TGV corrections.

|  | BP | Capon | | MUSIC | |
| --- | --- | --- | --- | --- | --- |
|  |  | UC | RC | UC | RC |
| UC | 0.77 | 0.82 | 0.78 | 0.72 | 0.79 |
| TGS | 0.92 | 0.78 | 0.85 | 0.69 | 0.78 |
| TGV | 0.90 | - | - | - | - |

**Table S4**:Accuracy analysis of the backscattered power at the 30 m layer-based AGB Models. *Here P* represents the backscattered power at the 30 m layer *layer*

| **Model** | **Polarimetric based models** | **BP** | | **Capon** | | **MUSIC** | |
| --- | --- | --- | --- | --- | --- | --- | --- |
|  |  | ***RMSE*** | ***r^2^*** | ***RMSE*** | ***r^2^*** | ***RMSE*** | ***r^2^*** |
| *P1* | $y=a_{0}+a_{1}P_{HH}$ | 21.96 | 0.75 | 29.47 | 0.56 | 27.36 | 0.62 |
| *P2* | $y=a_{0}+a_{1}P_{HV}$ | 17.07 | 0.85 | 23.32 | 0.72 | 27.41 | 0.62 |
| *P3* | $y=a_{0}+a_{1}P_{VV}$ | 23.24 | 0.73 | 26.57 | 0.64 | 26.50 | 0.64 |
| *P4* | $y=a_{0}+a_{1}P_{HH}+a_{2}P_{HH}^{2}$ | 23.19 | 0.75 | 29.25 | 0.57 | 24.95 | 0.68 |
| *P5* | $y=a_{0}+a_{1}P_{HV}+a_{2}P_{HV}^{2}$ | 16.60 | 0.86 | 21.93 | 0.76 | 25.67 | 0.67 |
| *P6* | $y=a_{0}+a_{1}P_{VV}+a_{2}P_{VV}^{2}$ | 23.75 | 0.73 | 24.79 | 0.69 | 25.90 | 0.67 |
| *P7* | $y=a_{0\cdot}\exp(a_{1}P_{HH})$ | 40.42 | 0.70 | 35.94 | 0.39 | 35.00 | 0.37 |
| *P8* | $y=a_{0\cdot}\exp(a_{1}P_{HV})$ | 42.20 | 0.81 | 41.96 | 0.52 | 35.20 | 0.37 |
| *P9* | $y=a_{0\cdot}\exp(a_{1}P_{VV})$ | 39.34 | 0.66 | 35.15 | 0.37 | 35.22 | 0.39 |
| *P10* | $y=a_{0}P_{HH}^{a_{1}}$ | 23.91 | 0.74 | 34.04 | 0.54 | 28.09 | 0.60 |
| *P11* | $y=a_{0}P_{HV}^{a_{1}}$ | 23.19 | 0.84 | 24.35 | 0.70 | 28.10 | 0.60 |
| *P12* | $y=a_{0}P_{VV}^{a_{1}}$ | 24.21 | 0.70 | 27.77 | 0.61 | 27.21 | 0.62 |
| *P13* | $y=a_{0}/{(a}_{1}+exp(-a_{2}P_{HH}))$ | 27.43 | 0.63 | 37.94 | 0.36 | 24.67 | 0.69 |
| *P14* | $y=a_{0}/{(a}_{1}+exp(-a_{2}P_{HV}))$ | 22.11 | 0.75 | 24.09 | 0.45 | 32.49 | 0.46 |
| *P15* | $y=a_{0}/{(a}_{1}+exp(-a_{2}P_{VV}))$ | 28.69 | 0.59 | 39.60 | 0.36 | 23.60 | 0.72 |

**Table S5**: The backscattered power at the 30 m layer-based AGB Models based on the combination of linear polarization. *Here P* represents the backscattered power at the 30 m layer *layer*

| **Model** | **Polarimetric based models** | **BP** | | **Capon** | | **MUSIC** | |
| --- | --- | --- | --- | --- | --- | --- | --- |
|  |  | ***RMSE*** | ***r^2^*** | ***RMSE*** | ***r^2^*** | ***RMSE*** | ***r^2^*** |
| *P16* | $y=a_{0}+{a_{1}P_{HV}+a}_{2}P_{VV}$ | 16.50 | 0.87 | 23.23 | 0.73 | 26.63 | 0.64 |
| *P17* | $y=a_{0}+{a_{1}P_{HV}+a}_{2}P_{HH}$ | 16.63 | 0. 86 | 19.60 | 0.81 | 26.88 | 0.63 |
| *P18* | $y=a_{0}+{a_{1}P_{HH}+a}_{2}P_{VV}$ | 22.29 | 0.75 | 26.10 | 0.66 | 30.18 | 0.65 |
| *P19* | $y=a_{0}+{a_{1}P_{HV}+a_{2}P_{HV}^{2}+a}_{3}P_{VV}+a_{4}P_{VV}^{2}$ | 16.14 | 0.87 | 22.84 | 0.75 | 26.40 | 0.66 |
| *P20* | $y=a_{0}+{a_{1}P_{HV}+a_{2}P_{HV}^{2}+a}_{3}P_{HH}+a_{4}P_{HH}^{2}$ | 16.86 | 0.86 | 16.66 | 0.86 | 25.35 | 0.68 |
| *P21* | $y=a_{0}+{a_{1}P_{HH}+a_{2}P_{HH}^{2}+a}_{3}P_{VV}+a_{4}P_{VV}^{2}$ | 20.96 | 0.78 | 22.14 | 0.76 | 25.50 | 0.68 |
| *P22* | $y=a_{0}+{a_{1}P_{HH}+a}_{2}P_{HV}+a_{3}P_{VV}$ | 16.27 | 0.86 | 19.69 | 0.81 | 26.64 | 0.64 |
| *P23* | $y=a_{0}+{a_{1}P_{HH}+a_{2}P_{HH}^{2}+a}_{3}P_{HV}+a_{4}P_{HV}^{2}+a_{5}P_{VV}+a_{6}P_{VV}^{2}$ | 16.27 | 0.86 | 19.69 | 0.82 | 26.64 | 0.64 |
| *P24* | $y=a_{0}+{a_{1}P_{HV}+a}_{2}{(P}_{HH}-P_{VV})$ | 17.25 | 0.85 | 21.75 | 0.76 | 27.82 | 0.60 |

**Table S6**:The backscattered power at the 30 m layer-based AGB is based on the combination of linear and non-linear polarizations. *Here P* represents the backscattered power at the 30 m layer *layer*

| **Model** | **Polarimetric based models** | **BP** | | **Capon** | | **MUSIC** | |
| --- | --- | --- | --- | --- | --- | --- | --- |
|  |  | ***RMSE*** | ***r^2^*** | ***RMSE*** | ***r^2^*** | ***RMSE*** | ***r^2^*** |
| *P25* | $y=a_{0}+{a_{1}P_{HH}+a}_{2}P_{HV}+a_{3}P_{PiH}$ | 14.31 | 0.90 | 19.74 | 0.80 | 26.91 | 0.63 |
| *P26* | $y=a_{0}+{a_{1}P_{VV}+a}_{2}P_{HV}+a_{3}P_{PiV}$ | 16.85 | 0.86 | 20.66 | 0.79 | 26.60 | 0.64 |
| *P27* | $y=a_{0}+{a_{1}P_{HH}+a}_{2}P_{HV}+a_{3}P_{RH}$ | 15.51 | 0.88 | 19.11 | 0.81 | 26.98 | 0.63 |
| *P28* | $y=a_{0}+{a_{1}P_{VV}+a}_{2}P_{HV}+a_{3}P_{RV}$ | 15.48 | 0.88 | 23.66 | 0.72 | 24.48 | 0.69 |
| *P29* | $y=a_{0}+{a_{1}P_{HH}+a}_{2}P_{VV}+a_{3}P_{RL}$ | 22.63 | 0.75 | 24.48 | 0.70 | 24.84 | 0.69 |
| *P30* | $y=a_{0}{{+a}_{1}P_{HH}+a_{2}P_{HH}^{2}+a}_{3}P_{HV}+a_{4}P_{HV}^{2}+a_{5}P_{PiH}+a_{6}P_{PiH}^{2}$ | 14.73 | 0.89 | 19.74 | 0.80 | 26.91 | 0.63 |
| *P31* | $y=a_{0}+{a_{1}P_{VV}+a_{2}P_{VV}^{2}+a}_{3}P_{HV}+a_{4}P_{HV}^{2}+a_{5}P_{PiV}+a_{6}P_{PiV}^{2}$ | 15.62 | 0.88 | 20.66 | 0.79 | 26.60 | 0.64 |
| *P32* | $y=a_{0}{{+a}_{1}P_{HH}+a_{2}P_{HH}^{2}+a}_{3}P_{HV}+a_{4}P_{HV}^{2}+a_{5}P_{RH}+a_{6}P_{RV}^{2}$ | 15.48 | 0.88 | 19.11 | 0.82 | 26.98 | 0.63 |
| *P33* | $y=a_{0}+{a_{1}P_{VV}+a_{2}P_{VV}^{2}+a}_{3}P_{HV}+a_{4}P_{HV}^{2}+a_{5}P_{RV}+a_{6}P_{RV}^{2}$ | 15.46 | 0.88 | 23.66 | 0.72 | 24.48 | 0.69 |
| *P34* | $y=a_{0}+a_{1}P_{HH}+a_{2}P_{HH}^{2}+a_{3}P_{VV}+a_{4}P_{VV}^{2}+a_{5}P_{RL}+a_{6}P_{RL}^{2}$ | 22.46 | 0.75 | 24.48 | 0.70 | 24.84 | 0.69 |

**Table S7**: Tomographic FH -based AGB Models. Here *H* represents FH retrieved from the TomoSAR cube estimated using BP, Capon, and MUSQC estimator.

| **Models** | **Height based models** | **BP** | | **Capon** | | **MUSIC** | |
| --- | --- | --- | --- | --- | --- | --- | --- |
|  |  | ***RMSE*** | ***r^2^*** | ***RMSE*** | ***r^2^*** | ***RMSE*** | ***r^2^*** |
| *H1* | $y=a_{0}+a_{1}H_{HH}$ | *36.63* | *0.31* | *31.48* | *0.51* | *36.77* | *0.37* |
| *H2* | $y=a_{0}+a_{1}H_{HV}$ | *23.14* | *0.72* | *28.01* | *0.61* | *28.69* | *0.59* |
| *H3* | $y=a_{0}+a_{1}H_{VV}$ | *26.82* | *0.63* | *30.83* | *0.63* | *34.82* | *0.5* |
| *H4* | $y=a_{0}+a_{1}H_{HH}+a_{2}H_{HH}^{2}$ | *33.48* | *0.41* | *31.18* | *0.51* | *36.88* | *0.37* |
| *H5* | $y=a_{0}+a_{1}H_{HV}+a_{2}H_{HV}^{2}$ | *23.06* | *0.73* | *26.48* | *0.64* | *27.31* | *0.63* |
| *H6* | $y=a_{0}+a_{1}H_{VV}+a_{2}H_{VV}^{2}$ | *25.80* | *0.66* | *30.25* | *0.54* | *34.45* | *0.47* |
| *H7* | $y=a_{0}exp(-a_{1}H_{HH})$ | *37.22* | *0.29* | *31.78* | *0.49* | *36.88* | *0.37* |
| *H8* | $y=a_{0}exp(-a_{1}H_{HV})$ | *23.77* | *0.71* | *28.66* | *0.59* | *29.38* | *0.57* |
| *H9* | $y=a_{0}exp(-a_{1}H_{VV})$ | *27.60* | *0.61* | *31.27* | *0.51* | *34.98* | *0.45* |
| *H10* | $y=a_{0}H_{HH}^{a_{1}}$ | *36.21* | *0.32* | *31.41* | *0.51* | *36.77* | *0.37* |
| *H11* | $y=a_{0}H_{HV}^{a_{1}}$ | *23.14* | *0.73* | *27.70* | *0.61* | *28.39* | *0.60* |
| *H12* | $y=a_{0}H_{VV}^{a_{1}}$ | *26.74* | *0.64* | *30.73* | *0.53* | *33.98* | *0.46* |
| *H13* | $y=a_{0}/{(a}_{1}+exp(-a_{2}H_{HH}))$ | *33.67* | *0.41* | *31.20* | *0.52* | *36.83* | *0.37* |
| *H14* | $y=a_{0}/{(a}_{1}+exp(-a_{2}H_{HV}))$ | *22.95* | *0.73* | *26.56* | *0.64* | *27.42* | *0.62* |
| *H15* | $y=a_{0}/{(a}_{1}+exp(-a_{2}H_{VV}))$ | *25.84* | *0.66* | *30.28* | *0.54* | *34.53* | *0.47* |

***Table S8****:* The backscattered power at the 30 m layer-based AGB *and tree height combined AGB Models. Here P* represents the backscattered power at the 30 m *layer, and H* represents FH retrieved from the TomoSAR cube estimated using BP, Capon, and MUSIC estimator.

| **Models** | **Combined models** | **BP** | | **Capon** | | **MUSIC** | |
| --- | --- | --- | --- | --- | --- | --- | --- |
|  |  | ***RMSE*** | ***r^2^*** | ***RMSE*** | ***r^2^*** | ***RMSE*** | ***r^2^*** |
| *C1* | $y=a_{0}+a_{1}P_{HH}+a_{2}H_{HH}$ | 20.01 | 0.81 | 25.28 | 0.67 | 24.36 | 0.70 |
| *C2* | $y=a_{0}+a_{1}P_{HV}+a_{2}H_{HV}$ | 13.82 | 0.90 | 20.72 | 0.78 | 23.60 | 0.72 |
| *C3* | $y=a_{0}+a_{1}P_{VV}+a_{2}H_{VV}$ | 15.95 | 0.87 | 21.34 | 0.77 | 21.27 | 0.77 |
| *C4* | $y=a_{0}+{a_{1}P_{HH}+a_{2}P_{HH}^{2}+a}_{3}H_{HH}+a_{4}H_{HH}^{2}$ | 22.87 | 0.79 | 24.58 | 0.69 | 23.65 | 0.72 |
| *C5* | $y=a_{0}+{a_{1}P_{HV}+a_{2}P_{HV}^{2}+a}_{3}H_{HV}+a_{4}H_{HV}^{2}$ | 12.23 | 0.92 | 17.87 | 0.84 | 22.13 | 0.75 |
| *C6* | $y=a_{0}+{a_{1}P_{VV}+a_{2}P_{VV}^{2}+a}_{3}H_{VV}+a_{4}H_{VV}^{2}$ | 17.47 | 0.85 | 20.19 | 0.79 | 22.40 | 0.75 |
| *C7* | $y={a_{0}\exp\left( -a_{1}P_{HH} \right)+a}_{2}\exp(-a_{3}H_{HH})$ | 23.50 | 0.75 | 26.34 | 0.65 | 24.97 | 0.68 |
| *C8* | $y={a_{0}\exp\left( -a_{1}P_{HV} \right)+a}_{2}\exp(-a_{3}H_{HV})$ | 15.05 | 0.88 | 21.94 | 0.76 | 24.50 | 0.70 |
| *C9* | $y={a_{0}\exp\left( -a_{1}P_{VV} \right)+a}_{2}\exp(-a_{3}H_{VV})$ | 16.87 | 0.86 | 22.56 | 0.74 | 21.99 | 0.75 |
| *C10* | $y=a_{0}+{a_{1}P_{HV}+a}_{2}P_{VV}+{a_{3}H}_{vv}$ | 13.00 | 0.91 | 20.97 | 0.78 | 21.44 | 0.77 |
| *C11* | $y=a_{0}+{a_{1}P_{HV}+a}_{2}P_{HH}+{a_{3}H}_{HV}$ | 14.20 | 0.90 | 19.44 | 0.81 | 23.54 | 0.72 |
| *C12* | $y=a_{0}+{a_{1}P_{HH}+a}_{2}P_{VV}+{a_{3}H}_{VV}$ | 15.35 | 0.88 | 21.53 | 0.77 | 21.95 | 0.76 |
| *C13* | $y=a_{0}+{a_{1}P_{HV}+a_{2}P_{HV}^{2}+a}_{3}P_{VV}+a_{4}P_{VV}^{2}+{a_{5}H}_{VV}+a_{6}H_{VV}^{2}$ | 14.04 | 0.90 | 20.44 | 0.79 | 22.13 | 0.76 |
| *C14* | $y=a_{0}+{a_{1}P_{HV}+a_{2}P_{HV}^{2}+a}_{3}P_{HH}+a_{4}P_{HH}^{2}+{a_{5}H}_{HV}+a_{6}H_{HV}^{2}$ | 13.97 | 0.90 | 14.40 | 0.87 | 22.91 | 0.74 |
| *C15* | $y=a_{0}+{a_{1}P_{HH}+a_{2}P_{HH}^{2}+a}_{3}P_{VV}+a_{4}P_{VV}^{2}+{a_{5}H}_{VV}+a_{6}H_{VV}^{2}$ | 16.33 | 0.88 | 20.44 | 0.80 | 24.43 | 0.72 |
| *C16* | $y=a_{0}+{a_{1}P_{HH}+a}_{2}P_{HV}+a_{3}P_{VV}+{a_{4}H}_{VV}$ | 13.59 | 0.91 | 19.42 | 0.81 | 21.76 | 0.76 |
| *C17* | $y=a_{0}+{a_{1}P_{HH}+a_{2}P_{HH}^{2}+a}_{3}P_{HV}+a_{4}P_{HV}^{2}+a_{5}P_{VV}+a_{6}P_{VV}^{2}+{a_{7}H}_{HV}+a_{8}H_{HV}^{2}$ | 15.85 | 0.88 | 18.2 | 0.84 | 23.98 | 0.73 |
| *C18* | $y=a_{0}+{a_{1}P_{HV}+a}_{2}{(P}_{HH}-P_{VV}){{+a}_{3}H}_{HV}$ | 13.21 | 0.91 | 19.83 | 0.80 | 25.33 | 0.68 |

**Table S9**: TomoSAR backscattered power metrics (Q1-Q5) derived AGB Models. Here $Q_{n}$ represents different TomoSAR metrics, and *H* represents tree height retrieved from the TomoSAR cube estimated using the BP estimator.

| **Model** | **Polarimetric based models** | **Q1** | | **Q2** | | **Q3** | | **Q4** | | **Q5** | |  |
| --- | --- | --- | --- | --- | --- | --- | --- | --- | --- | --- | --- | --- |
|  |  | ***RMSE*** | ***r^2^*** | ***RMSE*** | ***r^2^*** | ***RMSE*** | ***r^2^*** | ***RMSE*** | ***r^2^*** | ***RMSE*** | ***r^2^*** |  |
| *QP1* | | $y=a_{0}+a_{1}{Q_{n}}_{HH}$ | 42.21 | 0.09 | 43.96 | 0.07 | 43.28 | 0.05 | 35.86 | 0.33 | 44.12 | 0.05 |
| *QP2* | $y=a_{0}+a_{1}{Q_{n}}_{HV}$ | 45.22 | 0.009 | 47.88 | 0.001 | 43.78 | 0.02 | 20.90 | 0.81 | 45.61 | 0.02 |  |
| *QP3* | $y=a_{0}+a_{1}{Q_{n}}_{VV}$ | 44.59 | 0.01 | 48.44 | 0.00 | 44.11 | 0.01 | 33.69 | 0.42 | 45.78 | 0.01 |  |
| *QP4* | $y=a_{0}+a_{1}{Q_{n}}_{HH}+a_{2}{Q_{n}}_{HH}^{2}$ | 42.23 | 0.09 | 43.78 | 0.08 | 43.52 | 0.05 | 32.86 | 0.44 | 43.81 | 0.06 |  |
| *QP5* | $y=a_{0}+a_{1}{Q_{n}}_{HV}+a_{2}{Q_{n}}_{HV}^{2}$ | 44.01 | 0.02 | 42.82 | 0.08 | 42.91 | 0.06 | 16.28 | 0.87 | 44.32 | 0.05 |  |
| *QP6* | $y=a_{0}+a_{1}{Q_{n}}_{VV}+a_{2}{Q_{n}}_{VV}^{2}$ | 45.51 | 0.02 | 62.22 | 0.00 | 43.84 | 0.04 | 28.86 | 0.56 | 51.42 | 0.00 |  |
| *QP7* | $y=a_{0\cdot}\exp(a_{1}{Q_{n}}_{HH})$ | 42.00 | 0.09 | 42.56 | 0.07 | 42.97 | 0.05 | 36.88 | 0.31 | 43.25 | 0.05 |  |
| *QP8* | $y=a_{0\cdot}\exp(a_{1}{Q_{n}}_{HV})$ | 44.17 | 0.00 | 44.25 | 0.00 | 43.81 | 0.02 | 20.86 | 0.78 | 43.84 | 0.02 |  |
| *QP9* | $y=a_{0\cdot}\exp(a_{1}{Q_{n}}_{VV})$ | 43.90 | 0.01 | 44.22 | 0.00 | 44.02 | 0.01 | 34.49 | 0.39 | 44.06 | 0.01 |  |
| *QP10* | $y=a_{0}{Q_{n}}_{HH}^{a_{1}}$ | 42.00 | 0.09 | 42.56 | 0.07 | 42.97 | 0.05 | 36.88 | 0.31 | 43.25 | 0.05 |  |
| *QP11* | $y=a_{0}{Q_{n}}_{HV}^{a_{1}}$ | 44.17 | 0.00 | 44.25 | 0.00 | 43.81 | 0.02 | 20.86 | 0.78 | 43.84 | 0.02 |  |
| *QP12* | $y=a_{0}{Q_{n}}_{VV}^{a_{1}}$ | 43.90 | 0.01 | 44.22 | 0.00 | 44.02 | 0.01 | 34.48 | 0.39 | 44.06 | 0.01 |  |
| *QP13* | $y=a_{0}/{(a}_{1}+exp(-a_{2}{Q_{n}}_{HH}))$ | 41.99 | 0.09 | 42.50 | 0.08 | 43.07 | 0.05 | 36.48 | 0.32 | 43.15 | 0.05 |  |
| *QP14* | $y=a_{0}/{(a}_{1}+exp(-a_{2}{Q_{n}}_{HV}))$ | 44.16 | 0.00 | 44.25 | 0.00 | 43.98 | 0.02 | 23.22 | 0.73 | 43.93 | 0.02 |  |
| *QP15* | $y=a_{0}/{(a}_{1}+exp(-a_{2}{Q_{n}}_{VV}))$ | 43.86 | 0.01 | 44.28 | 0.00 | 44.17 | 0.00 | 33.64 | 0.42 | 44.12 | 0.01 |  |
| *QP16* | $y=a_{0}+{a_{1}{Q_{n}}_{HV}+a}_{2}{Q_{n}}_{VV}$ | 44.06 | 0.02 | 44.93 | 0.00 | 41.01 | 0.14 | 17.48 | 0.85 | 44.69 | 0.01 |  |
| *QP17* | $y=a_{0}+{a_{1}{Q_{n}}_{HV}+a}_{2}{Q_{n}}_{HH}$ | 40.28 | 0.19 | 43.62 | 0.08 | 38.40 | 0.25 | 18.20 | 0.85 | 44.72 | 0.04 |  |
| *QP18* | $y=a_{0}+{a_{1}{Q_{n}}_{HH}+a}_{2}{Q_{n}}_{VV}$ | 40.45 | 0.18 | 45.91 | 0.05 | 42.12 | 0.24 | 33.47 | 0.43 | 44.39 | 0.04 |  |
| *QP19* | $y=a_{0}+{a_{1}{Q_{n}}_{HV}+a_{2}{Q_{n}}_{HV}^{2}+a}_{3}{Q_{n}}_{VV}+a_{4}{Q_{n}}_{VV}^{2}$ | 44.98 | 0.05 | 45.63 | 0.09 | 40.54 | 0.21 | 15.83 | 0.88 | 46.35 | 0.03 |  |
| *QP20* | $y=a_{0}+{a_{1}{Q_{n}}_{HV}+a_{2}{Q_{n}}_{HV}^{2}+a}_{3}{Q_{n}}_{HH}+a_{4}{Q_{n}}_{HH}^{2}$ | 39.27 | 0.23 | 36.92 | 0.29 | 43.32 | 0.20 | 19.00 | 0.85 | 37.47 | 0.33 |  |
| *QP21* | $y=a_{0}+{a_{1}{Q_{n}}_{HH}+a_{2}{Q_{n}}_{HH}^{2}+a}_{3}{Q_{n}}_{VV}+a_{4}{Q_{n}}_{VV}^{2}$ | 40.61 | 0.19 | 42.01 | 0.13 | 33.52 | 0.45 | 28.56 | 0.59 | 42.89 | 0.10 |  |
| *QP22* | $y=a_{0}+{a_{1}{Q_{n}}_{VV}+a_{2}{Q_{n}}_{VV}^{2}+a}_{3}{Q_{n}}_{HV}+a_{4}{Q_{n}}_{HV}^{2}+a_{5}{Q_{n}}_{PiV}+a_{6}{Q_{n}}_{PiV}^{2}$ | 42.28 | 0.11 | 45.68 | 0.01 | 38.88 | 0.23 | 17.73 | 0.84 | 43.57 | 0.06 |  |
| *QP23* | $y=a_{0}{{+a}_{1}{Q_{n}}_{HH}+a_{2}{Q_{n}}_{HH}^{2}+a}_{3}{Q_{n}}_{HV}+a_{4}{Q_{n}}_{HV}^{2}+a_{5}{Q_{n}}_{RH}+a_{6}{Q_{n}}_{RV}^{2}$ | 37.29 | 0.29 | 42.09 | 0.12 | 39.85 | 0.23 | 16.99 | 0.85 | 36.67 | 0.33 |  |
| *QP24* | $y=a_{0}+{a_{1}{Q_{n}}_{HV}+a}_{2}{(Q_{n}}_{HH}-{Q_{n}}_{VV})$ | 41.13 | 0.14 | 43.17 | 0.05 | 40.92 | 0.15 | 19.07 | 0.82 | 43.23 | 0.05 |  |

**Table S10** Combined FH and TomoSAR metrics (Q1-Q5) derived AGB Models. Here $Q_{n}$ represents different TomoSAR metrics, and *H* represents FH estimated from the TomoSAR cube of the BP estimator.

| **Models** | **Combined models** | **Q1** | | **Q2** | | **Q3** | | **Q4** | | **Q5** | |
| --- | --- | --- | --- | --- | --- | --- | --- | --- | --- | --- | --- |
|  |  | ***RMSE*** | ***r^2^*** | ***RMSE*** | ***r^2^*** | ***RMSE*** | ***r^2^*** | ***RMSE*** | ***r^2^*** | ***RMSE*** | ***r^2^*** |
| *QC1* | $y=a_{0}+a_{1}{Q_{n}}_{HH}+a_{2}H_{HH}$ | 34.42 | 0.40 | 37.19 | 0.29 | 35.86 | 0.35 | 33.45 | 0.44 | 37.46 | 0.29 |
| *QC2* | $y=a_{0}+a_{1}{Q_{n}}_{HV}+a_{2}H_{HV}$ | 37.92 | 0.27 | 38.05 | 0.26 | 38.55 | 0.24 | 19.47 | 0.81 | 38.55 | 0.25 |
| *QC3* | $y=a_{0}+a_{1}{Q_{n}}_{VV}+a_{2}H_{VV}$ | 35.73 | 0.35 | 36.43 | 0.33 | 36.00 | 0.34 | 30.19 | 0.54 | 36.60 | 0.32 |
| *QC4* | $y=a_{0}+{a_{1}{Q_{n}}_{HH}+a_{2}{Q_{n}}_{HH}^{2}+a}_{3}H_{HH}+a_{4}H_{HH}^{2}$ | 37.61 | 0.55 | 32.59 | 0.44 | 36.42 | 0.47 | 37.93 | 0.30 | 32.29 | 0.46 |
| *QC5* | $y=a_{0}+{a_{1}{Q_{n}}_{HV}+a_{2}{Q_{n}}_{HV}^{2}+a}_{3}H_{HV}+a_{4}H_{HV}^{2}$ | 15.75 | 0.88 | 29.77 | 0.76 | 19.97 | 0.80 | 14.49 | 0.89 | 19.95 | 0.84 |
| *QC6* | $y=a_{0}+{a_{1}{Q_{n}}_{VV}+a_{2}{Q_{n}}_{VV}^{2}+a}_{3}H_{VV}+a_{4}H_{VV}^{2}$ | 21.47 | 0.77 | 26.17 | 0.68 | 21.62 | 0.76 | 24.41 | 0.70 | 26.14 | 0.68 |
| *QC7* | $y={a_{0}\exp\left( -a_{1}{Q_{n}}_{HH} \right)+a}_{2}exp(-a_{3}H_{HH})$ | 19.26 | 0.81 | 18.86 | 0.81 | 22.80 | 0.74 | 22.10 | 0.78 | 20.20 | 0.79 |
| *QC8* | $y={a_{0}\exp\left( -a_{1}{Q_{n}}_{HV} \right)+a}_{2}exp(-a_{3}H_{HV})$ | 34.64 | 0.38 | 38.42 | 0.25 | 36.27 | 0.33 | 37.10 | 0.30 | 37.66 | 0.27 |
| *QC9* | $y={a_{0}\exp\left( -a_{1}{Q_{n}}_{VV} \right)+a}_{2}exp(-a_{3}H_{VV})$ | 23.38 | 0.72 | 25.51 | 0.66 | 25.36 | 0.67 | 26.67 | 0.64 | 26.69 | 0.64 |
| *QC10* | $y=a_{0}+{a_{1}{Q_{n}}_{HV}+a}_{2}{Q_{n}}_{VV}+{a_{3}H}_{vv}$ | 36.34 | 0.33 | 36.20 | 0.34 | 33.90 | 0.42 | 17.22 | 0.85 | 37.28 | 0.30 |
| *QC11* | $y=a_{0}+{a_{1}{Q_{n}}_{HV}+a}_{2}{Q_{n}}_{HH}+{a_{3}H}_{HV}$ | 35.25 | 0.37 | 35.91 | 0.35 | 34.68 | 0.39 | 17.40 | 0.85 | 38.80 | 0.24 |
| *QC12* | $y=a_{0}+{a_{1}{Q_{n}}_{HH}+a}_{2}{Q_{n}}_{VV}+{a_{3}H}_{VV}$ | 35.11 | 0.38 | 35.61 | 0.36 | 34.15 | 0.41 | 34.74 | 0.40 | 36.72 | 0.32 |
| *QC13* | $y=a_{0}+{a_{1}{Q_{n}}_{HV}+a_{2}{Q_{n}}_{HV}^{2}+a}_{3}{Q_{n}}_{VV}+a_{4}{Q_{n}}_{VV}^{2}+{a_{5}H}_{VV}+a_{6}H_{VV}^{2}$ | 38.28 | 0.34 | 37.38 | 0.38 | 36.83 | 0.39 | 17.92 | 0.85 | 37.43 | 0.39 |
| *QC14* | $y=a_{0}+{a_{1}{Q_{n}}_{HV}+a_{2}{Q_{n}}_{HV}^{2}+a}_{3}{Q_{n}}_{HH}+a_{4}{Q_{n}}_{HH}^{2}+{a_{5}H}_{HV}+a_{6}H_{HV}^{2}$ | 36.27 | 0.38 | 35.83 | 0.38 | 37.26 | 0.37 | 19.24 | 0.82 | 34.72 | 0.43 |
| *QC15* | $y=a_{0}+{a_{1}{Q_{n}}_{HH}+a_{2}{Q_{n}}_{HH}^{2}+a}_{3}{Q_{n}}_{VV}+a_{4}{Q_{n}}_{VV}^{2}+{a_{5}H}_{VV}+a_{6}H_{VV}^{2}$ | 38.04 | 0.33 | 37.42 | 0.36 | 27.68 | 0.64 | 40.59 | 0.34 | 40.14 | 0.26 |
| *QC16* | $y=a_{0}+{a_{1}{Q_{n}}_{HH}+a}_{2}{Q_{n}}_{HV}+a_{3}{Q_{n}}_{VV}+{a_{4}H}_{VV}$ | 32.93 | 0.45 | 34.60 | 0.40 | 32.19 | 0.48 | 17.20 | 0.85 | 37.71 | 0.29 |
| *QC17* | $y=a_{0}+{a_{1}{Q_{n}}_{HH}+a_{2}P_{HH}^{2}+a}_{3}{Q_{n}}_{HV}+a_{4}P_{HV}^{2}+a_{5}{Q_{n}}_{VV}+a_{6}{Q_{n}}_{VV}^{2}+{a_{7}H}_{HV}+a_{8}H_{HV}^{2}$ | 35.90 | 0.44 | 37.75 | 0.39 | 28.85 | 0.63 | 21.02 | 0.80 | 38.56 | 0.80 |
| *QC18* | $y=a_{0}+{a_{1}{Q_{n}}_{HV}+a}_{2}{(Q_{n}}_{HH}-{PQ_{n}}_{VV}){{+a}_{3}H}_{HV}$ | 35.55 | 0.36 | 35.71 | 0.36 | 35.14 | 0.38 | 25.95 | 0.66 | 36.63 | 0.32 |

**Supplementary Figure**


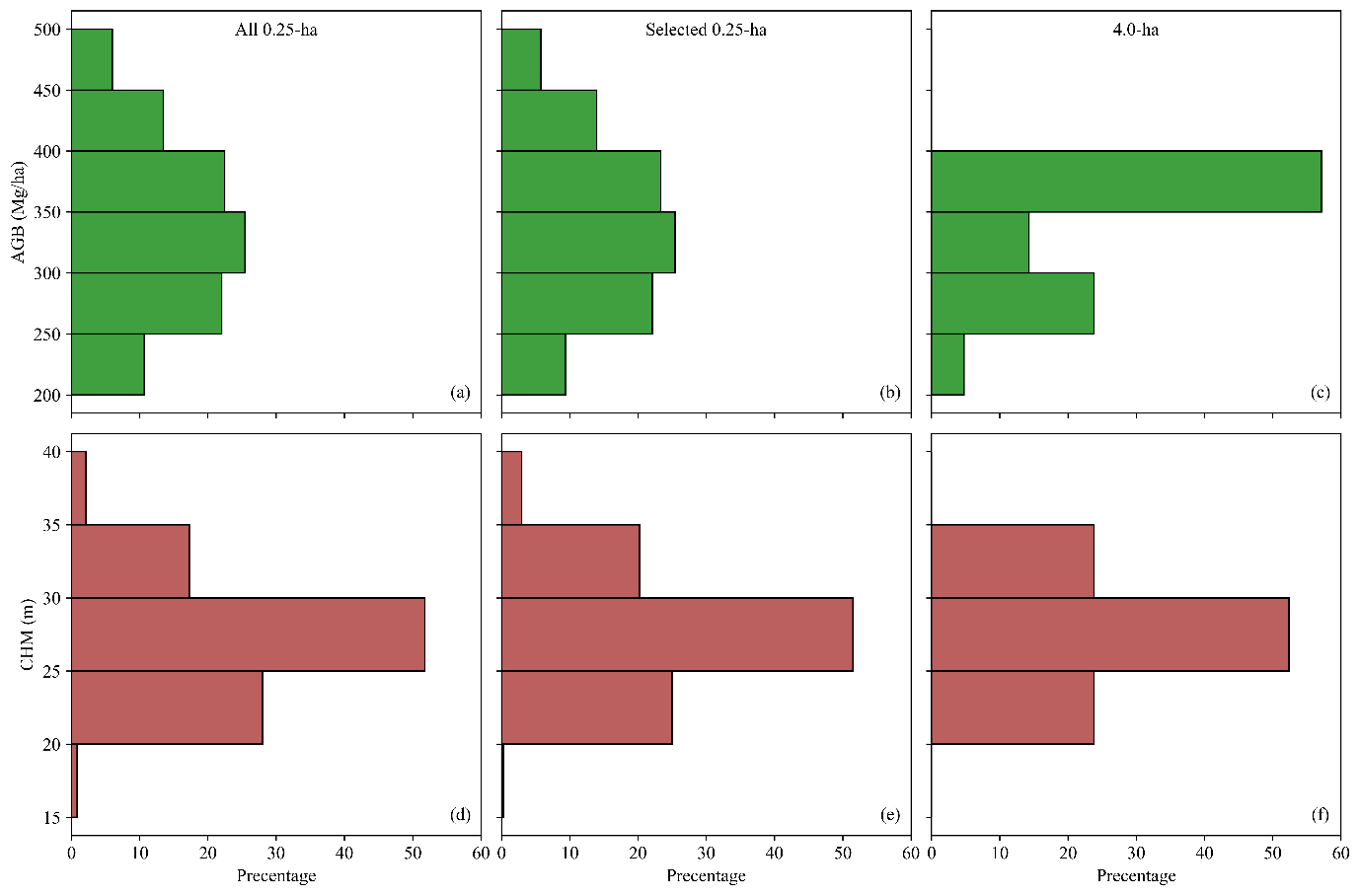


Fig. S1: The distribution of AGB (a-c) and FH (d-f) in all 0.25-ha plots, selected 0.25-ha plots used for generating 4-ha plots, and 4-ha plots


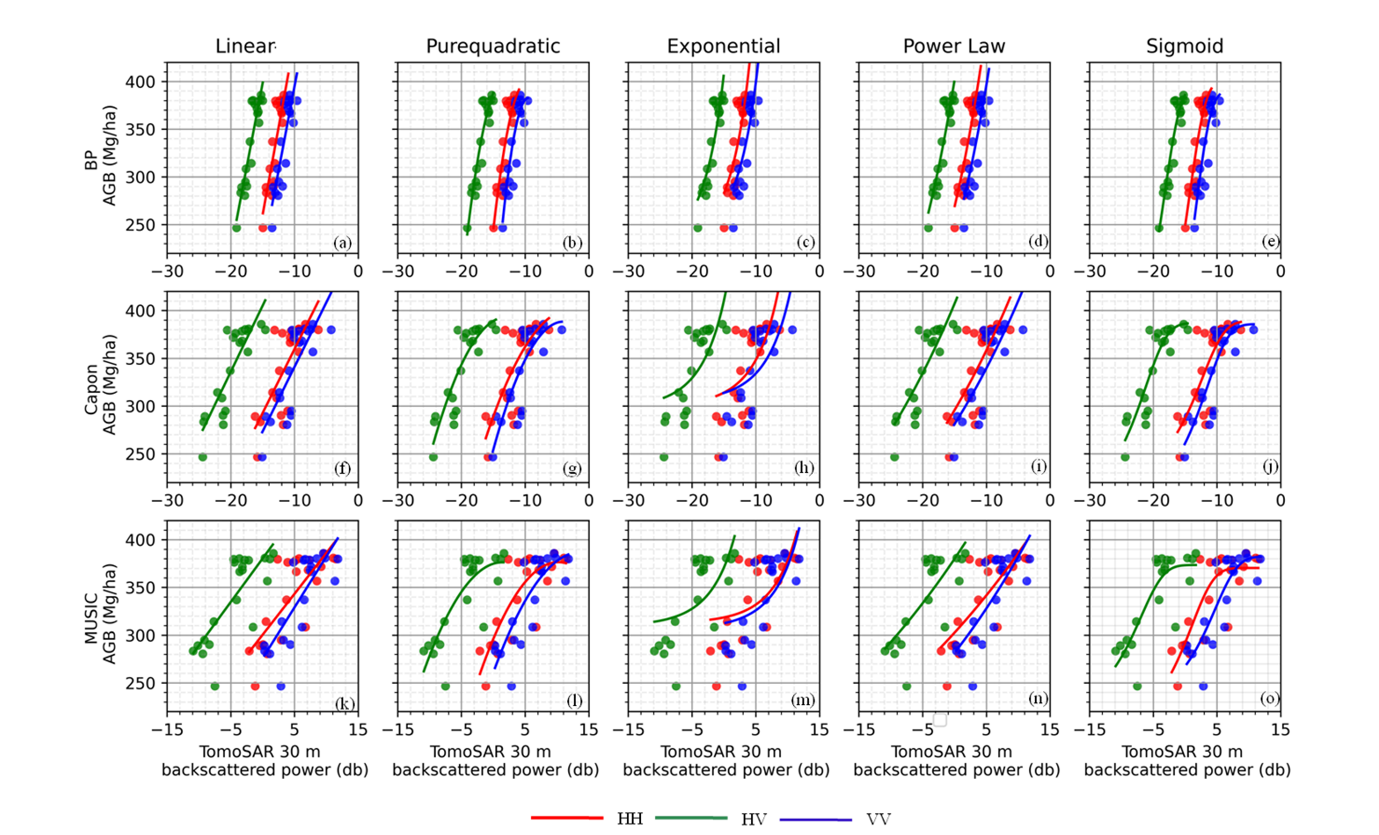
Fig. S2: The relationship between TomoSAR 30 m layer backscattered power and AGB was established using linear, pure-quadratic, exponential, power law, and sigmoid form for BP (a-e), Capon (f-j), and MUSIC (k-o) estimators.


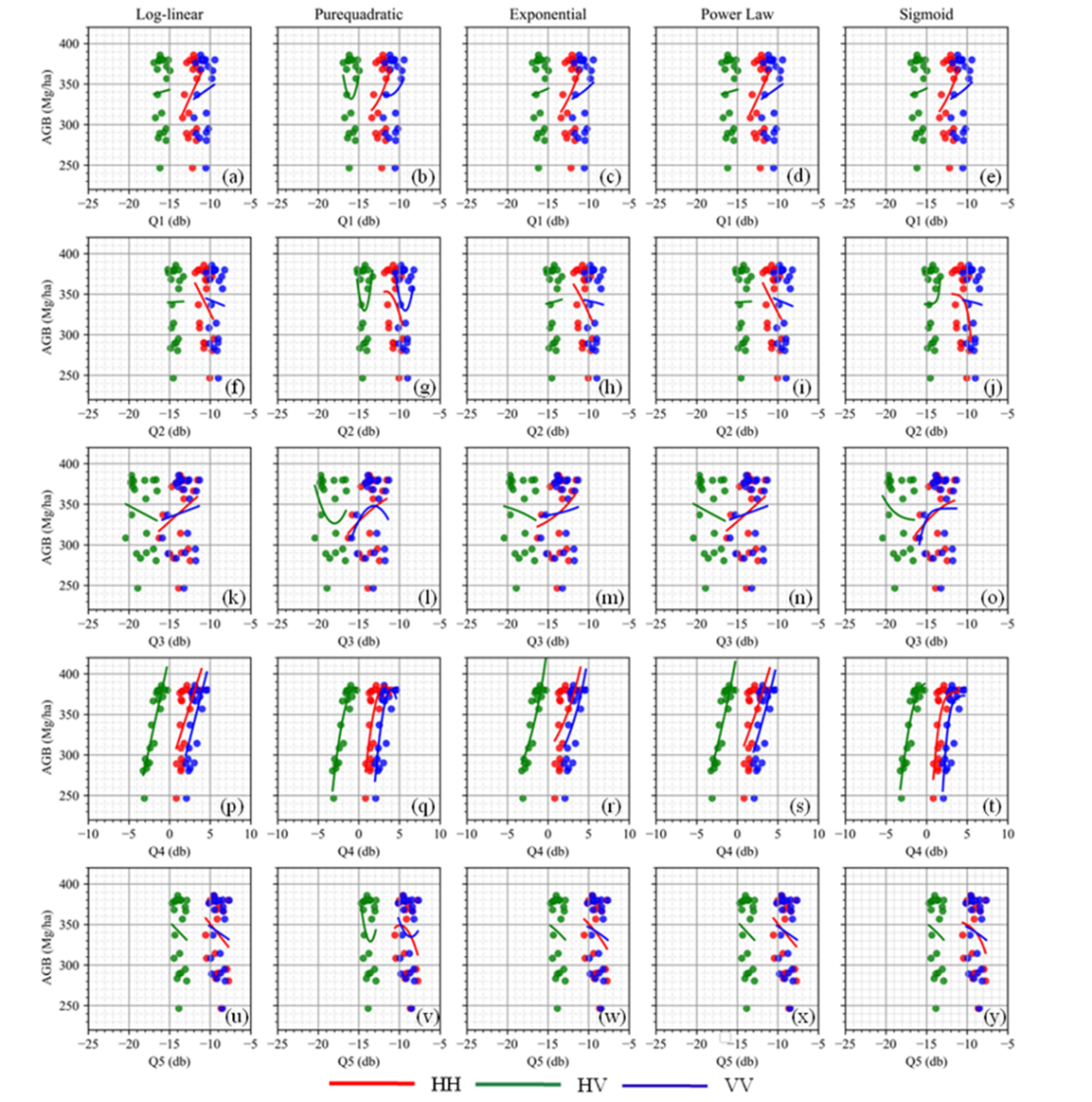


Fig. S3: The relationship between TomoSAR backscattered power metric *Q1* (a-e), *Q2* (f-j), *Q3* (k-o), *Q4* (p-t), *Q5* (u-y), and AGB established using linear, pure-quadratic, exponential, power law and sigmoid form for BP estimators.

References

1. Reigber, A. & Moreira, A. First demonstration of airborne SAR tomography using multibaseline L-band data. *IEEE Trans. Geosci. Remote Sensing* **38,** 2142–2152 (2000).

2. Ramachandran, N., Saatchi, S., Tebaldini, S., d’Alessandro, M. M. & Dikshit, O. Evaluation of P-Band SAR Tomography for Mapping Tropical Forest Vertical Backscatter and Tree Height. *Remote Sensing* **13,** 1485 (2021).
